# An investigation of the seasonal relationships between meteorological factors, water quality, and sporadic cases of Legionnaires’ disease in Washington, DC

**DOI:** 10.1017/S0950268823000651

**Published:** 2023-05-15

**Authors:** Alexander Kirpich, Aleksandr Shishkin, Pema Lhewa, Chen Yang, Michael E. von Fricken, Michael H. Norris, Thomas A. Weppelmann

**Affiliations:** 1 Department of Population Health Sciences, School of Public Health, Georgia State University, Atlanta, GA, USA; 2 Department of Biostatistics, Harvard T.H. Chan School of Public Health, Harvard University, Boston, MA, USA; 3 Department of Global and Community Health, College of Public Health, George Mason University, Fairfax, VA, USA; 4 Department of Geography, University of Florida, Gainesville, FL, USA; 5 Emerging Pathogens Institute, University of Florida, Gainesville, FL, USA; 6 Department of Internal Medicine, University of South Florida, Tampa, FL, USA

**Keywords:** Legionnaires’, disease, environmental water quality, drinking water quality, meteorological factors, seasonality, Washington, DC

## Abstract

Since the discovery of Legionnaires’ disease (LD), limited progress has been made in understanding the epidemiology of sporadic cases of LD. Outbreaks have confirmed that air conditioning and potable water systems can be sources of community-acquired LD. However, studying the association between water quality and LD incidence has been challenging due to the heterogeneity of water systems across large geographic areas. Furthermore, although seasonal trends in incidence have been linked to increased rainfall and temperatures, the large geographic units have posed similar difficulties. To address this issue, a retrospective ecological study was conducted in Washington, DC, from 2001 to 2019. The study identified aseasonal pattern of LD incidence, with the majority of cases occurring between June and December, peaking in August, October, and November. Increased temperature was found to be associated with LD incidence. In surface water, higher concentrations of manganese, iron, and strontium were positively associated with LD, while aluminum and orthophosphate showed a negative association. Intreatment plant water, higher concentrations of total organic carbon, aluminum, barium, and chlorine were positively associated with LD, while strontium, zinc, and orthophosphate showed a negative association. The results for orthophosphates and turbidity were inconclusive, indicating the need for further research.

## Introduction

Originally discovered in 1976 at a convention of the American Legion in Philadelphia, legionellosis is caused by either pneumonic or non-pneumonic infections with Gram-negative *Legionella* spp. [1]. Unlike the localised outbreak of 1976, which led to its discovery, the majority of LD cases are acquired within communities where corresponding common point source(s) are not always properly identified. Those sources may include human-created environments such as air conditioning systems, fountains, wastewater, ice/ice machines, room humidifiers, mist machines, etc., as well as natural reservoirs such as surface water, groundwater, and rainwater [2,3]. In particular, *Legionella* spp. was isolated from a wide range of environments including rivers, lakes, puddles, and moist soil, as well as from municipal water distribution systems, hospital water supplies, and hotel water supplies [4–6].

For example within the natural reservoirs and aquatic environments, *Legionella pneumophila* is found in either biofilms or within their natural protozoan predators. As a result, their location within biofilms and intracellular niches leads to higher resistance to sterilising chemicals and environmental pressures [7–11]. For example, *Legionella pneumophila* that persist in amoebae have higher nutrient uptake, increased aquatic fitness, and an increased ability to infect mammalian cells [4]. The associations of the meteorological factors with LD cases have been studied and confirmed multiple times [12]. Within human-created environments, *Legionella* spp. has been found in various water supply systems, including water towers [9,13–16]. In these environments, *Legionella* spp. Are known to replicate within protozoan hosts and persist in biofilms [17–19].

The potential amplification of *Legionella* spp. in municipal water may pose a serious public health risk. This happens because human infection most commonly occurs due to inhaling *Legionella*-containing aerosols from contaminated water sources [17], while direct human-to-human transmission is known to be rare [20]. After inhalation by humans, it can be engulfed by macrophages in the alveolar parts of the lungs. The bacteria replicate within the macrophages and can cause a severe type of pneumonia known as Legionnaires’ disease [17,21]. Individuals at higher risk for developing Legionnaires’ disease are older individuals 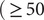 years) who have predisposing risk factors, such as smoking habits, chronic cardiovascular or respiratory disease, diabetes, alcoholism, and immunosuppression [17,22,23]. In the United States, during the past twenty years (i.e., 2000 and onwards), there has been a continuous *and* steady increase in LD cases. In particular, in 2018, more than a five-fold increase in LD cases per 100,000 was documented in comparison to the early 2000s [24,25].

At the same time, the incidence of LD cases has not been spatially uniform. For example, geographic heterogeneity was observed in the early 2000s when a large increase in LD was observed in the Mid-Atlantic Region [26]. Multiple causes have been hypothesised, such as the increasing median age of Americans raising the proportion of the elderly population, better diagnostic tests, more effective surveillance, and increased testing of pneumonia patients for severe acute respiratory syndrome (SARS) infections in early 2003. Those explanations, however, failed to explain much of the temporal or geographic variations in the incidence at that time [27].

Historically, due to the relatively low frequency of reported LD cases in small administrative units, previous epidemiological analyses relied on aggregates of cases at the regional level (i.e., the large geographic area) [12,27–32]. Studies addressing the association of LD cases with municipal water quality and climate have the potential to be unreliable and are challenging to conduct due to case aggregation over administrative units and corresponding heterogeneity in water sources and climatic environments over specified large geographic areas [12,33,34].

To overcome the challenge of large geographic units, the discrete municipality of Washington, DC, was selected for this retrospective ecological study from 2001 to 2019 because of the following reasons: i) it is *located* in the Northeastern Mid-Atlantic region which had a history of outbreaks earlier; ii) it is geographically *homogenous* and is located within the same climatic region (i.e., humid subtropical climate) with precise meteorological data; iii) it has had only a *single source of municipal water* drawn from the Potomac River, and, *most importantly*; iv) it has maintained detailed water quality records of both surface water and water processed by treatment plants. As a result, those detailed data records make this LD incidence study possible on a more precise and very detailed scale.

In summary, this study aimed i) to ascertain if changes in municipal or surface water component concentrations were associated with the increase in sporadic cases of LD and ii) to ascertain whether meteorological factors and climatic changes [12] as well as monthly seasonality were associated with the increase in sporadic cases of LD.

## Methods

### Incidence, meteorological, and water quality data sources

LD (Legionnaires’ disease) is a reportable disease in the United States [35]. Weekly counts of Legionnaires’ disease cases in Washington, DC, from January 2001 to December 2019 were obtained from the Centers for Disease Control and Prevention (CDC) Notifiable Infectious Disease Data Tables [36]. In particular, the CDC reports include cases which are clinically compatible and have laboratory-confirmed infections [37]. This includes both Legionnaires’ disease, which is typically characterised by fever with pneumonia, as well as Pontiac fever, which only has mild clinical symptoms without pneumonia. Water quality measurements were obtained from the United States Army Corps of Engineers (USACE) Water Analysis Reports [38]. The measurements included records for *both* the *untreated* surface water of the Potomac River and the *treated* water from two facilities (Dalecarlia and McMillan Treatment Plants) that serve as Washington, DC, treatment sources. The monthly records from January 2001 to December 2019 analyzed in this study include multiple organic and inorganic measurements available monthly. The majority of studied water components have previously been studied in relation to health and selected because of that reason. The surface water measurements include pH, concentrations of nitrate [39–43], orthophosphate [44–48], manganese, strontium [49], barium [50–55], nickel, total organic carbon [56], turbidity [56–59], aluminium [60–63], zinc [60], and iron. The treated water measurements include pH, concentrations of nitrate [39–43], orthophosphate [44–48], manganese, strontium [49], barium [50–55], nickel, total organic carbon, turbidity [56–59], aluminium [60–63], total coliform [64], heterotrophic plate count (HPC), zinc [60], iron, and chlorine [65–68]. Meteorological data were obtained from the National Oceanographic and Atmospheric Association (NOAA) in the form of the Global Summary of the Day (GSOD) generated reports. Since the Ronald Reagan International Airport monitoring station (WBAN ID: 72405013743) is directly adjacent to Washington, DC, the data from that station [69,70] were used for the analysis. In particular, the obtained meteorological daily measurements included *total* precipitation (i.e., rain and/or melted snow) in inches (in), WDSP – mean wind speed (WDSP) in knots (knots), and *average* temperature in Fahrenheit (°F). The GSOD measurements were available daily for the entire studied period from January 2001 to December 2019.

### Data processing

The available data were thoroughly checked and minor discrepancies were fixed before the analysis. In particular, those weeks that had no reporting of LD (i.e., coded as ‘-’ symbol which denotes ‘No reported cases’ and as the symbol ‘N’ which denotes ‘Not reportable’ cases) received zero counts. The reported weekly counts (from 1 to 52) within each year were cumulative and therefore non-decreasing from week to week. Those cumulative counts were used to compute the corresponding weekly increments for each week. For the year 2008, the presumably cumulative (and non-decreasing) counts decreased from 7 to 3 from week 20 to week 21 [71,72], and counts started to be non-decreasing again from 3. Since no explanation was available for this 7 to 3 move, the increase from week 20 to 21 was assumed to be 0, and cumulative counting proceeded further within 2008 starting from 3 in week 21, so that those weekly increments were properly counted. For the year 2012, the last reported week was 23 [73] and the cumulative reporting stopped afterwards until the end of the year. Those non-reported weekly values from week 24 were coded as 0. For water treatment facilities, if the measurement was available for both plants, then the average was taken as the final measurement, while if the measurement was available for one plant only (i.e., due to plant repairs or missing data), then that value was taken as the final measurement. The averaged values for each compound for the two plants were taken since i) the measurements between the plants varied only slightly [38] and ii) there was no way to detect which of the reported cases used the water from which plant so the averages were used as proxies. The values of not detected (‘ND’) reported for treatment plants for the compounds were coded as 0. The final sets of considered water measurement variables together with the corresponding units were summarised in Supplementary Tables S1 (river water) and S2 (treatment plant water). The meteorological measurements from the weather station were converted to the International System of Units (also known as the SI system) during the study period, which resulted in temperature being converted to degrees in Celsius (C), WDSP being converted to metres per second (m/s), and precipitation being converted to centimetres (cm). In the end, all the data were processed to be on the same time scale across all datasets. In particular, the weekly case counts were aggregated monthly, the daily meteorological measurements were averaged monthly for temperature and wind speed, and summed monthly for precipitation.

### Statistical analysis

In total, three different sets of potential predictors were considered for the statistical modelling of LD cases. Those sets of predictors were selected since for the majority of them, their relationship and association with *Legionella* spp. or LD cases have previously been considered or studied. The first set included meteorological factors (temperature, precipitation, and WDSP) [12], together with the categorical variable month to account for seasonality, as the set of predictors. The second set included meteorological factors (temperature, precipitation, and WDSP) [12] and surface water characteristics such as pH, concentrations of nitrate, orthophosphate, manganese, strontium, barium, nickel, total organic carbon, turbidity, aluminium, zinc, and iron as predictors. The third set included meteorological factors (temperature, precipitation, and WDSP) [12] and treatment plant water characteristics such as pH, concentrations of nitrate, orthophosphate, manganese, strontium, barium, nickel, total organic carbon, turbidity, aluminium, total coliform, heterotrophic plate count (HPC), zinc, iron, and chlorine as predictors.

The Poisson and negative binomial generalised linear modelling frameworks were used for the preliminary analysis [74–76]. Those models were determined to be insufficient in capturing the data variability for the given data due to excess zero counts. To address that concern, hurdle versions of Poisson and negative binomial models were used [77]. In the hurdle model, there are two components: the *binary* hurdle component, which accounts for excessive zeroes in the distribution (i.e., it models zeroes vs. non-zeroes) with the logit link; and the *truncated* Poisson (or negative binomial) distribution, which is used for the remaining counts starting from one (i.e., omitting zero) with the log link. Both components have the same set of predictors, but the coefficient estimates for those predictors are different. Those components are subsequently referred to as the *logit component* or the hurdle component of the model and the *log component* or the counts component of the model, respectively. The interpretation of the model coefficients is performed accordingly, that is, for the logit component, they are identical to the logistic regression for zero vs non-zero counts, while for the log component, they are the same as for the Poisson or negative binomial model with a log link but restricted to non-zero counts, within the model. The *logit component* fit is identical for both models, while the *log component* fits may differ since the negative binomial model provides more flexible fits than the Poisson model [77]. The interpretation of the model’s coefficients is provided in terms of the estimated odds for the *logit component* and in terms of the expected increases or decreases in counts based on the multipliers which are made from the exponentiated values of the estimated predictor coefficients for the *log component.* Since the month is a categorical variable, the first month (January) is used as the baseline (i.e., the reference for the other months) during the model-fitting process due to the structure of the model. As a result, there is no estimate for January in the model fit output. All data processing, statistical analyses, and figures were produced in R programming language [78] and RStudio [79], while package pscl [80] was used for the hurdle model fitting. The source code files together with the processed datasets were made publicly available on GitHub and can be downloaded [81].

## Results

### Summary of cases

**Figure 1.**
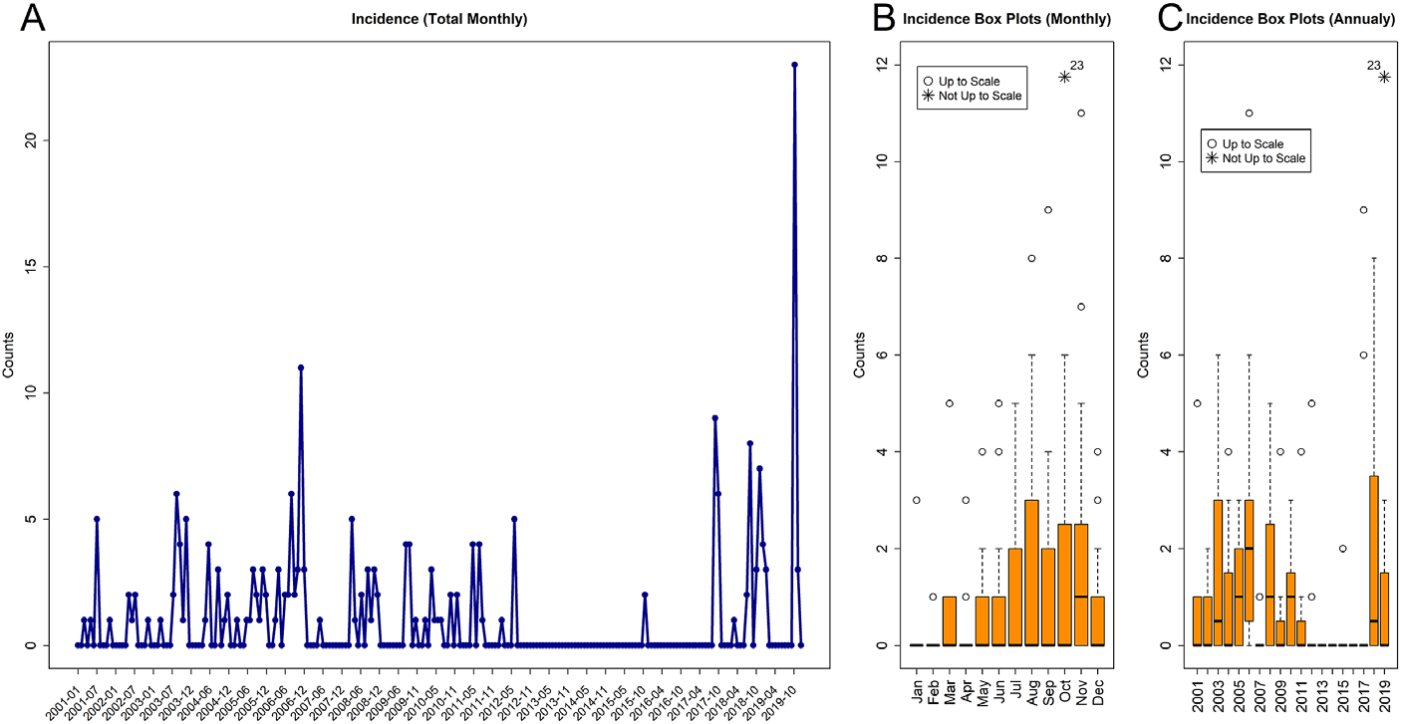
The visualisation of the case counts for each studied month (panel a) is reported together with the box and whisker plots both *monthly* across all studied years (panel b) and *yearly* across all months within each year (panel c).

The raw summaries of case counts for each month over time are provided in Figure 1a. The same raw counts are summarised as box and whisker plots both *monthly* across all studied years (Figure 1b) and *yearly* across all months within each year (Figure 1c). Based on panels a, b, and c of Figure 1, large heterogeneity of counts was observed with the majority of cases happening from August to November, while the largest number of cases was observed in the early 2000s and in 2019.

If the entire set of raw case counts is desired, it is presented in Table 1 for all months and for all studied years. The total number of months was 228, while the number of months with any reported LD cases was 71. The total number of cases during those months was 215. The total number of months without any reported cases was 157.

**Table 1.** Monthly reports of Legionnaires’ disease cases for Washington, DC, from January 2001 until December 2019

Year	Jan	Feb	Mar	Apr	May	Jun	Jul	Aug	Sep	Oct	Nov	Dec
2001	0	0	1	0	1	0	5	0	0	0	1	0
2002	0	0	0	0	2	1	2	0	0	0	1	0
2003	0	0	1	0	0	0	2	6	4	1	5	0
2004	0	0	0	0	1	4	0	0	3	0	1	2
2005	0	0	1	0	0	1	1	3	2	1	3	2
2006	0	0	1	3	0	2	2	6	2	3	11	3
2007	0	0	0	0	1	0	0	0	0	0	0	0
2008	0	0	5	1	0	2	0	3	1	3	2	0
2009	0	0	0	0	0	0	0	4	4	0	1	0
2010	0	1	0	3	1	1	1	0	0	2	0	2
2011	0	0	0	0	4	0	4	1	0	0	0	0
2012	0	1	0	0	0	5	0	0	0	0	0	0
2013	0	0	0	0	0	0	0	0	0	0	0	0
2014	0	0	0	0	0	0	0	0	0	0	0	0
2015	0	0	0	0	0	0	0	0	0	0	2	0
2016	0	0	0	0	0	0	0	0	0	0	0	0
2017	0	0	0	0	0	0	0	0	9	6	0	0
2018	0	0	1	0	0	0	2	8	0	3	7	4
2019	3	0	0	0	0	0	0	0	0	23	3	0

The inspection of Table 1 and Figure 1 reveals the annual seasonality of cases, with almost no cases in the first quarter of the year during the colder winter months and most of the cases in the third and fourth quarters (late summer and early fall). The summaries of cases for each month across all studied years are provided in Table 2. In particular, the median value for all (except November) months was 0, while for November it was 1, and the 75% percentile values across all months were between 0 and 3. The largest observed number of cases during any month was 42 in October. The monthly summaries also indicate the rarity of the LD incidence. There were no records found regarding the excessive counts of October 2019 or any earlier counts that were linked to any single outbreak [82], and so the counts were analyzed in a routine way with the rest of the data.

**Table 2.** Monthly summaries of Legionnaires’ disease cases with the corresponding quantiles across studied years for Washington, DC, from January 2001 until December 2019

Month	Mean	St. dev.	Min (0%)	Lw.Qrtl. (25%)	Median (50%)	Up.Qrtl. (75%)	Max (100%)	Total
Jan	0.16	0.69	0	0	0	0	3	3
Feb	0.11	0.32	0	0	0	0	1	2
Mar	0.53	1.17	0	0	0	1	5	10
Apr	0.37	0.96	0	0	0	0	3	7
May	0.53	1.02	0	0	0	1	4	10
Jun	0.84	1.46	0	0	0	1	5	16
Jul	1	1.49	0	0	0	2	5	19
Aug	1.63	2.59	0	0	0	3	8	31
Sep	1.32	2.33	0	0	0	2	9	25
Oct	2.21	5.3	0	0	0	2.5	23	42
Nov	1.95	2.91	0	0	1	2.5	11	37
Dec	0.68	1.25	0	0	0	1	4	13

The analogous summaries for each year summarised across all months within that year are provided in Table 3. In particular, the median value varied from 0 to 1 across all years, while the 75% percentile values varied from 0 to 3.25 across all years. The largest observed number of cases during the year was 33 in 2006. Those summaries also indicate the rarity of the LD incidence.

**Table 3.** Annual summaries of Legionnaires’ disease cases with the corresponding quantiles across all months within each year for Washington, DC, from January 2001 until December 2019

Month	Mean	St. Dev.	Min (0%)	Lw.Qrtl. (25%)	Median (50%)	Up.Qrtl. (75%)	Max (100%)	Total
2001	0.67	1.44	0	0	0	1	5	8
2002	0.5	0.8	0	0	0	1	2	6
2003	1.58	2.19	0	0	0.5	2.5	6	19
2004	0.92	1.38	0	0	0	1.25	4	11
2005	1.17	1.11	0	0	1	2	3	14
2006	2.75	3.11	0	0.75	2	3	11	33
2007	0.08	0.29	0	0	0	0	1	1
2008	1.42	1.62	0	0	1	2.25	5	17
2009	0.75	1.54	0	0	0	0.25	4	9
2010	0.92	1	0	0	1	1.25	3	11
2011	0.75	1.54	0	0	0	0.25	4	9
2012	0.5	1.45	0	0	0	0	5	6
2013	0	0	0	0	0	0	0	0
2014	0	0	0	0	0	0	0	0
2015	0.17	0.58	0	0	0	0	2	2
2016	0	0	0	0	0	0	0	0
2017	1.25	2.99	0	0	0	0	9	15
2018	2.08	2.87	0	0	0.5	3.25	8	25
2019	2.42	6.58	0	0	0	0.75	23	29

The other useful summaries are the pairwise relationships between the considered predictors of interest and for the *logit and log components* of the model are summarised in Figure 2 for meteorological factors and in Supplementary Figures S1–S28 for the studied water components.

**Figure 2.**
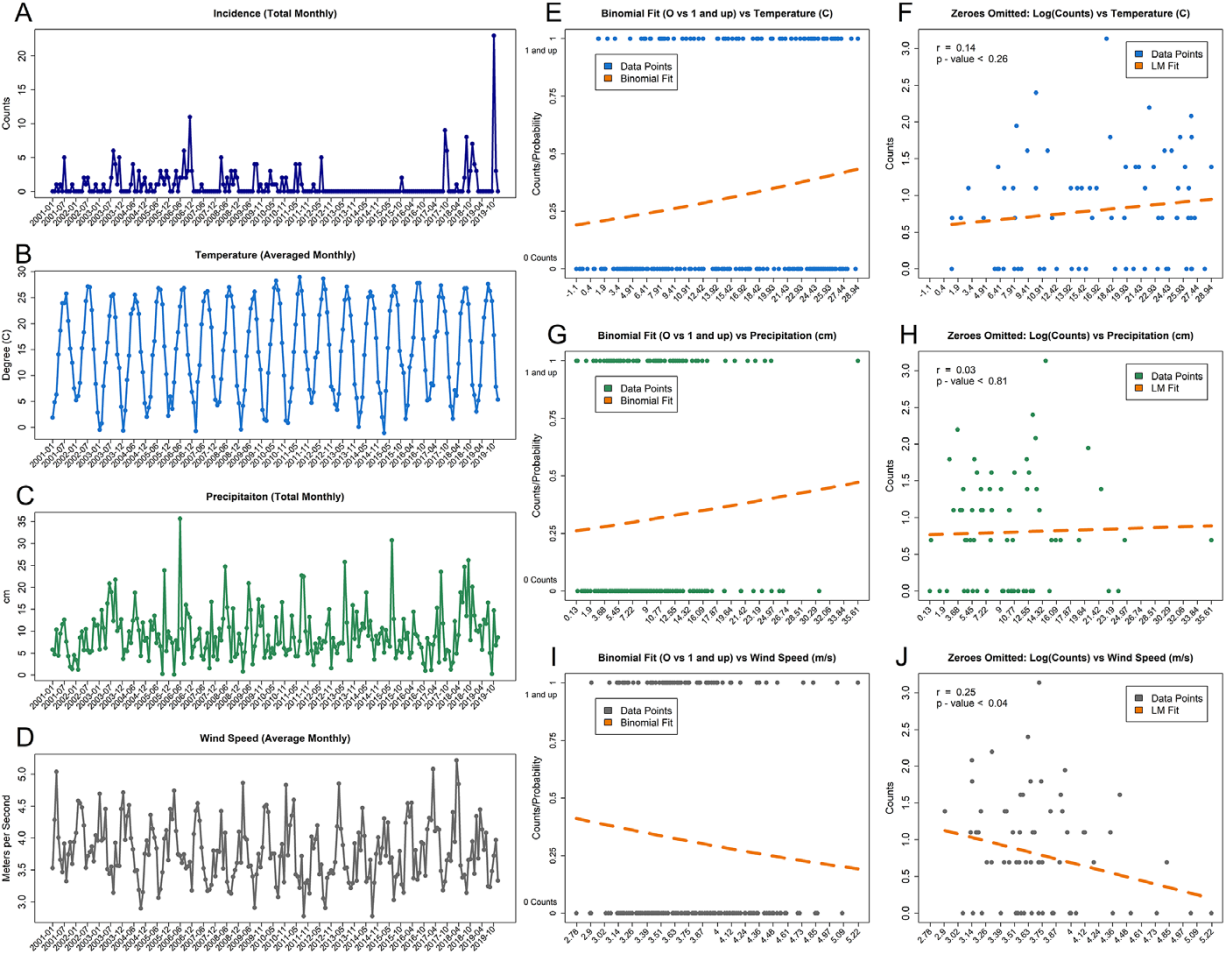
The visualisation of the LD case counts for each studied month (panel a) reported together with the environmental predictors such as average temperature (panel b), total precipitation (panel c), and the average wind speed (panel d). The corresponding pairwise relationships for the pairwise logit components (panels e, g, and i) and log components (panels f, h, and j) are also provided. For the logit components (panels e, g, and i), the logistic regression probability fits of zero vs non-zero counts based on the corresponding predictors are displayed in dark orange (Binomial Fit). For the log components (panels f, h, and j), the linear model fits of log non-zero counts based on the corresponding predictor are displayed in dark orange (LM Fit).

More precisely, since the model has two components (logit for zero vs non-zero counts and log for the non-zero counts), the corresponding *pairwise relationships* between each predictor and the transformed case counts can be illustrated visually. Such relationships are presented for meteorological factors (temperature, precipitation, and wind speed) in Figure 2 panels e, g, and i for the *logit component* and in panels f, h, and j for the *log component.* The raw data for the LD case counts and the meteorological predictors are presented in panels a–d. The analogous figures for the river predicting compounds and for the treatment plant water predicting compounds are presented in panels c and d of Supplementary Figures S1–S28 along with the raw data in panels a and b. In summary, the binomial fits in the presented figures visualise the *logit (hurdle) component* relationships of the data and the model and the *log (counts) component* relationships of the data and the model, respectively.

The detailed summaries of the monthly predictor’s ranges for all investigated parameters for the *river (surface) water* model are presented in Tables 4 and 5, while the detailed summaries of the monthly predictor’s ranges of all investigated parameters for the *treatment plant water* model are presented in Tables 6 and 7. The 2.4% to 97.6% quantiles contain 95% of the observed values for a given predictor and summarise the corresponding range of the predictor’s values. The 50% percentile represents the median value for a given predictor.

**Table 4.** The summary statistics over all months from January 2001 until December 2019 for the predictors used in the *surface water model* together with the corresponding measurement units

Statistic	Temperature (C)	Precipitation (cm)	Wind Speed (m/s)	pH	Nitrate (ppm)	Orthophosphate (ppm)	Manganese (ppb)	Strontium (ppb)
0%	−1.1	0.13	2.78	6.9	0	0	10	58
2.40%	0.39	1.2	3.04	7.3	0.41	0	17.5	87.9
25%	7.08	5.42	3.43	7.7	1.2	0	34	127.75
50%	14.76	8.27	3.73	7.8	1.6	0	44	157
75%	23.7	12.04	4.08	8	2	0	66.25	201.5
97.60%	27.51	24.34	4.84	8.4	2.6	0.33	174.92	269.76
100%	28.94	35.61	5.22	8.8	3.04	0.77	354	311
Mean	14.91	9.31	3.78	7.85	1.56	0.04	57.44	167.24
St.Dev.	8.7	5.73	0.47	0.28	0.56	0.1	43.94	49.86

**Table 5.** The summary statistics over all months from January 2001 until December 2019 for the predictors used in the *surface water model* together with the corresponding measurement units

Statistic	Barium (ppb)	Nickel (ppb)	Total Organic Carbon (ppb)	Turbidity (NTU)	Aluminium (ppb)	Total Coliform (MPN/100mL)	Zinc (ppb)	Iron (ppb)
0%	17.1	0	1.39	1	23	5	0	0
2.40%	31	0.9	1.9	2	101.27	17	0	58.9
25%	35	1.2	2.5	5	204	718	2	111.75
50%	38.1	1.7	2.82	9	264.5	2531.5	2.9	167
75%	43.28	2.3	3.4	15	363.25	5641.25	4	318
97.60%	53	3.76	4.45	40.55	1066.07	39721.57	8.87	1373.78
100%	72	6.4	5.1	65	2389	97250	58	3245
Mean	39.72	1.82	2.95	12.21	334.18	5866.02	3.46	288.2
St.Dev.	6.27	0.82	0.67	10.25	269.63	12055.31	4.12	368.33

**Table 6.** The summary statistics over all months from January 2001 until December 2019 for the predictors used in the *treatment plant water model* together with the corresponding measurement units

Statistic	Temperature (C)	Precipitation (cm)	Wind Speed (m/s)	pH ()	Nitrate (ppm)	Orthophosphate (ppm)	Manganese (ppb)	Strontium (ppb)	Barium (ppb)
0%	−1.1	0.13	2.78	7.65	0	0	0	98	26.5
2.40%	0.39	1.2	3.04	7.7	0.37	0	0.4	109	29.5
25%	7.08	5.42	3.43	7.7	1.15	2.3	0.65	134	32.5
50%	14.76	8.27	3.73	7.7	1.5	2.4	0.8	160.75	35.35
75%	23.7	12.04	4.08	7.7	1.9	2.45	1.2	200.62	39.28
97.60%	27.51	24.34	4.84	8.4	2.56	3.2	6.01	267.93	46.5
100%	28.94	35.61	5.22	8.45	3.01	3.34	30.7	306	53.75
Mean	14.91	9.31	3.78	7.77	1.51	2.01	1.39	170.71	36.14
St.Dev.	8.7	5.73	0.47	0.18	0.56	1	2.84	44.81	4.73

**Table 7.** The summary statistics over all months from January 2001 until December 2019 for the predictors used in the *treatment plant water model* together with the corresponding measurement units

Statistic	Nickel (ppb)	Total Organic Carbon (ppb)	Turbidity (NTU)	Aluminium (ppb)	Total Coliform (Positive) (MPN/100mL)	Heterotrophic Plate Count (CFU/mL)	Zinc (ppb)	Iron (ppb)	Chlorine (ppm)
0%	0	1.15	0.01	15	0	0.5	0	0	3
2.40%	0.6	1.21	0.01	17	0	1	0	0	3.2
25%	0.85	1.55	0.03	27	0	1	0.64	0	3.65
50%	1.05	1.8	0.04	36	0	1.5	1	0	3.7
75%	1.91	2	0.06	50	0	9.12	1.51	0	3.71
97.60%	2.5	2.38	0.08	95.88	0.4	47	4.89	37.1	3.8
100%	6.75	2.65	0.09	472	0.45	266	14.25	212	3.8
Mean	1.36	1.78	0.04	42.93	0.02	10.57	1.31	6.16	3.66
St.Dev.	0.7	0.31	0.02	35.6	0.08	28.29	1.61	21.68	0.13

If the same summaries are desired individually for each year and are summarised for all months within that year and for each month summarised across all studied years, they are available as separate tables in the Supplementary material in the form of individual files for each year and month, respectively.

### Initial analysis: Meteorological factors and seasonality with LD incidence

The complete model outputs for the hurdle Poisson and hurdle negative binomial models for the association of LD incidence with temperature (°C), precipitation (cm), wind speed (m/s), and monthly seasonality (for each month in relation to the baseline month of January) are presented in the Supplementary Files S1 and S2. *Only statistically significant results* 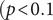 ) are summarised in this section, while the entire model outputs are provided in the Supplementary material.

For the *logit component* (which is identical for the two models), the baseline odds of having non-zero counts versus zero counts for zero values of all other predictors was estimated to be 0.004 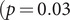 ), confirming the rarity of the Legionnaires’ disease in general. The meteorological factors were not significant for the presence of any counts vs zero counts, while the last three months of the year had increased odds of LD incidence in comparison to the baseline month of January. More precisely, the odds of LD case presence were estimated to be 18.719 times 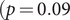 ) *higher* for October, 34.377 times 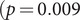 ) *higher* for November, and 8.168 times 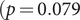 ) *higher* for December, than the baseline month of January.

For the *log component,* results differed for the Poisson model and for the negative binomial model. For the negative binomial model, no statistically significant associations were detected. For the Poisson model, *given the counts are positive* for every temperature unit increase (C), the estimated *increase* in case counts is 1.181 times 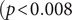 ) *higher* than without such an increase in temperature. In a similar way, given the counts are positive, there is an estimated decrease in case counts for being in March 0.186 times 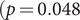 ) *lower*, in April 0.105 times 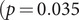 ) *lower*, in May 0.027 times 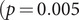 ) *lower*, in June 0.021 times 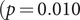 ) *lower*, in July by 0.016 times 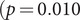 ) *lower*, in August 0.039 times 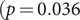 ) *lower* and in September by 0.061 times 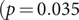 ) *lower* in comparison to the baseline month of January.

Within the *single* *-paragraph* *summary* of the meteorological factors and the seasonality models, there are indications that the months of October, November, and December were estimated to be positively associated with any presence of LD cases vs no cases in relation to the baseline month of January. Also, given that cases are present, there are indications that the *increase* in the number of cases is associated with the increase in temperature, while the *decrease* in the number of cases is expected in the months of March, April, May, June, July, August, and September.

### Surface water analysis: Meteorological factors and water with LD incidence

The complete model outputs for the hurdle Poisson and hurdle negative binomial models for the association of LD incidence with temperature (°C), precipitation (cm), wind speed (m/s), pH, nitrate (ppm), orthophosphate (ppm), manganese (ppb), strontium (ppb), barium (ppb), nickel (ppb), total organic carbon (ppb), turbidity (NTU), aluminium (ppb), zinc (ppb), and iron (ppb) are presented in the Supplementary Files S3 and S4. *Only statistically significant results* 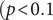 ) are summarised in this section, while the entire model outputs are provided in the Supplementary material.

For the *logit component* (which is identical for the two models), the meteorological factors were not significant for the presence of any counts vs zero counts.

For the *logit component* (which is identical for the two models), some statistically significant associations were identified for the river water components (manganese, aluminium, and iron). In particular, the odds of LD cases presence for manganese were estimated to be 1.015 times 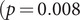 ) *higher*, for aluminium were estimated to be 0.994 times 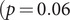 ) *lower*, and for iron were estimated to be 1.003 times 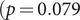 ) *higher* for the corresponding unit increase in the mentioned compounds. The corresponding predictor’s units and ranges are summarised in Tables 4 and 5.

For the *log component,* the meteorological factors results differed for the Poisson model and for the negative binomial model. For the Poisson model *given the counts are positive* for every temperature unit increase (°C), the expected *decrease* in case counts is estimated to be 0.965 times 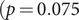 ), while for every wind speed unit increase (m/s), the expected *decrease* in case counts is estimated to be 0.540 times 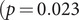 ). For the negative binomial model *given the counts are positive* only for every wind speed unit increase (m/s), the expected *decrease* in case counts is estimated to be 0.310 times 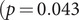 ).

For the *log component,* different statistically significant associations for the Poisson and for the negative binomial were also identified for the river water components. For the Poisson model *given the counts are positive* for every orthophosphate unit increase (ppm), the expected *decrease* in case counts is estimated to be 0.071 times 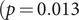 ), for every strontium unit increase (ppb) the expected *increase* in case counts is estimated to be 1.009 times 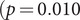 ), while for every aluminium unit increase (ppb) the expected *decrease* in case counts is estimated to be 0.998 times 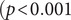 ). No stable estimate was produced for the iron component by the Poisson model. For the negative binomial model *given the counts are positive* only for every orthophosphate unit increase (ppm), the expected *decrease* in case counts is estimated to be 0.024 times 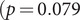 ).

Within the *single-paragraph summary* of the meteorological factors and the surface water components models, there are indications that an increase in the concentration of manganese and iron is positively associated and of aluminium is negatively associated with the presence of LD cases in any amounts. Also *given that cases are present* for the meteorological factors, there are indications that a further increase in the number of cases is associated with the *decrease* of temperature and wind speed. For the river water compounds, *given that cases are present*, the increase in concentrations of orthophosphate, and aluminium are associated with the decrease in the number of cases, while the increase in the concentration of strontium is associated with the increase in the number of cases.

### Treatment plants water: Meteorological factors and water with LD incidence

The complete model outputs for the hurdle Poisson and hurdle negative binomial models for the association of LD incidence with temperature (°C), precipitation (cm), wind speed (m/s), pH, nitrate (ppm), orthophosphate (ppm), manganese (ppb), strontium (ppb), barium (ppb), nickel (ppb), total organic carbon (ppb), turbidity (NTU), aluminium (ppb), total coliform-positive (MPN/100mL), heterotrophic plate count (CFU/mL), Zinc (ppb), Iron (ppb), Chlorine (ppm) are presented in the Supplementary Files S5 and S6. *Only statistically significant results* 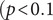 ) are summarised in this section, while the entire model outputs are provided in the Supplementary material.

For the *logit component* (which is identical for the two models), the baseline odds of having non-zero counts versus zero counts for zero values of all predictors was estimated to be <0.001 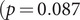 ) confirming the rarity of Legionnaires’ disease in general. In the meantime, for the *logit component,* no meteorological factors were statistically significant for the presence of any counts vs zero counts.

For the *logit component* (which is identical for the two models), some statistically significant associations were identified for the treatment plant water components (strontium, total organic carbon, turbidity, aluminium, zinc and chlorine). In particular, the odds of LD cases presence for strontium were estimated to be 0.978 times 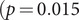 ) *lower*, for total organic carbon were estimated to be 5.250 times 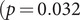 ) *higher*, for turbidity were estimated to be ~4.77 x 10^26^ times 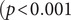 ) *higher*, for aluminium were estimated to be 1.013 times 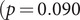 ) *higher*, for zinc were estimated to be 0.664 times 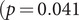 ) *lower*, and for chlorine were estimated to be 2100.687 times 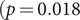 ) *higher* for the corresponding unit increase in the mentioned compounds. The corresponding predictor’s units and ranges are summarised in Tables 6 and 7.

For the *log component,* the meteorological factors results were similar for the Poisson model and for the negative binomial model with no statistically significant associations.

For the *log component,* different statistically significant associations for the Poisson and for the negative binomial were also identified for the treatment plant components. For the Poisson model, *given the counts are positive* for every nitrate unit increase (ppm), the expected *decrease* in case counts is estimated to be 0.513 times 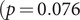 ); for every orthophosphate unit increase (ppm), the expected *decrease* in case counts is estimated to be 0.736 times 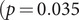 ); for every barium unit increase (ppb), the expected *increase* in case counts is estimated to be 1.103 times 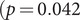 ); for every turbidity unit increase (NTU), the expected *decrease* in case counts is estimated to be <0.001 times 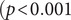 ); while for every chlorine unit increase (ppm), the expected *increase* in case counts is estimated to be 4962.582 times 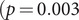 ). For the negative binomial model, *given the counts are positive* for every orthophosphate unit increase (ppm), the expected *decrease* in case counts is estimated to be 0.690 times 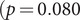 ); for every turbidity unit increase (NTU), the expected *decrease* in case counts is estimated to be <0.001 times 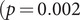 ); while for every chlorine unit increase (ppm), the expected *increase* in case counts is estimated to be 10572.286 times 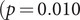 ).

In summary of the meteorological factors and the treatment plant water models, no meteorological factors for either *logit component* or *log component* of any of the models were statistically significant for the presence of any case counts or increase in case counts. There were indications, however, that the increase in concentrations of total organic carbon, turbidity, aluminium, and chlorine is *positively* associated with the presence of LD cases in any amounts, while the increase in concentration of strontium and zinc is *negatively* associated with the presence of LD cases in any amounts. For the treatment plant water quality measurements, *given that cases are present*, the increase in concentrations of barium and chlorine is associated with the *increase* in the number of cases, while the increase in the concentration of nitrate, orthophosphate, and turbidity is associated with the *decrease* in the number of cases.

## Discussion

### Raw data summaries

The annual seasonality of LD cases identified in the present study partially corroborates the earlier CDC observations that the highest annual frequency of cases occurs around the late summer and early fall [83]. In particular, when specific months were investigated (Table 2), the largest monthly averages and the total monthly counts (across all years) were observed from July to November. This was confirmed in the meteorological factors and the seasonality models where the months of October, November, and December were statistically significant and associated with the presence of LD cases in any amount. At the same time, the observed seasonality *has not been consistent* from year to year (Table 3), when the largest numbers of cases were reported from 2003 to 2006 and from 2017 to 2019 and no cases were reported during 2013 and 2014. Within a given year, the most frequent monthly presence was observed during 2006 when cases were reported in eight months during the year, with the most cases during the last two quarters. We hypothesise that the inconsistencies in predictions from year to year are linked to the sparsity of LD outbreaks during some years.

### Incidence data reporting quality

While there can be multiple explanations for the observed heterogeneity, among the most obvious ones is the potential underreporting. In particular, the symptoms of Legionnaires’ disease are not specific, that is, it presents as a range of clinical manifestations and symptoms which may cause *misdiagnosis* of the pathogen and subsequent undercounting of LD cases [84–87]. More specifically, the myriad of symptoms associated with LD such as pneumonia, hyponatremia, hypophosphatemia, increased liver enzyme levels, acute mental status changes, headache, diarrhoea, early onset of pleuritic pain, diarrhoea, and fever with multisystem disease including rhabdomyolysis with renal failure can contribute to the difficulty of a differential diagnosis by medical practitioners [22,88]. The CDC reports, which were used for the analysis, include all clinically compatible and laboratory-confirmed cases [37]. Therefore, the cases can either be Legionnaires’ disease with pneumonia or Pontiac fever, which only has mild clinical symptoms. While underreporting of some level is expected for both disease manifestations, the Pontiac fever infections are hypothesised to have much smaller testing rates and to comprise only a small fraction of all reported cases, if any. This is expected to happen as more severe infections are more likely to be investigated thoroughly and clinically tested. There is no way, however, from the available CDC reporting data to distinguish between the two.

At the same time, for LD infection, the time to detection is critical, especially for high-risk populations. Nowadays several methods can be used to detect LD, including serological and antibody-based assays, bacterial culture, urinary antigen tests, and real-time PCR. Within this set of available tests, not all of them may always have the desired sensitivity, which varies by method [89]. For example, serology tests as well as the urinary antigen tests for LD infections have different issues [90]. Recently PCR-based methods have become more common and are now regarded as the best molecular method for detection because they offer specificity, sensitivity, and desired speed [17,22].

The Notifiable Infectious Disease Data Tables [36], which were used for the analysis, had some data discrepancies for 2008 and 2012, which were discussed earlier. It is also important to point out that based on the available data, it is not possible to completely exclude potential travel-associated LD. In particular, cases reported in the Disease Data Tables [36] for Washington, DC, may have consumed water or spent time outside Washington, DC, due to frequent commutes and domestic or international travels. The sparsity of data (Tables 1–3) and the low counts for each individual month can be a source of instability for the model’s fits and the corresponding estimates. In particular, the estimates for iron concentrations were unstable (Supplementary Files S3).

### Meteorological factors and seasonality with LD incidence

There was an agreement between the empirical observations of the reported LD in any amount in the last three months of a given year and the statistical significance of the corresponding estimates. There has been a notable difference between the meteorological factors and seasonality model results for the Poisson and for the negative binomial models, *given that cases are present* for a few months in the middle of the year. In particular, *given that cases are present*, the months of March through September had negative statistically significant associations in comparison to the reference January month for the Poisson version of the hurdle model. Importantly, *given that cases are present,* the increase in temperature was *positively associated* with the increase in LD cases for a Poisson model. This can be explained mathematically by the fact that the Poisson hurdle model is more restrictive and less versatile than the corresponding negative binomial analogue. Overall, the meteorological and seasonality models confirmed the presence of monthly seasonality for cases as well as an indication of positive associations with the temperature increase and the corresponding LD incidence, given the cases are detected.

Aside from pronounced monthly seasonal patterns of LD incidence, the identified association of higher temperatures with an increase in incidence has biologically plausible explanations. In particular, elevated water temperatures have been known to increase the occurrence of *L. pneumophila* and a range of its thermophilic host organisms [4]. Since the trophozoite forms of the free-living amoebae that support intracellular replication of *L. pneumophila* have a growth optimum between 20 and 50 °C and the *L. pneumophila* virulence regulator *csrA*, which allows for intracellular replication, is only expressed above 25 °C, it is likely that the pathogen density in the aquatic environment is highest during the warm summer months [91]. It is also plausible that resources are more available due to decreases in microbial diversity and abundance within the aquatic community during warmer temperatures, which allow for higher nutrient availability that supports the growth of protozoa containing intracellular *L. pneumophila* [92,93]. In a similar way the potential decrease in microbial diversity in treated water due to an increase in chlorine levels may lead to higher nutrient availability. This availability of nutrients could increase the presence of *L. pneumophila* and lead to the corresponding observed increase in incidence.

Even though precipitation has been previously demonstrated to alter the distribution of various host organisms for *L. pneumophila* in the aquatic environment and has the potential to simultaneously increase both organic and inorganic nutrients that lead to the proliferation of *L. pneumophila* in surface water [94,95], such association with the precipitation level was not identified from the data. More specifically, there were no direct indications from the data that the increased monthly precipitation has been associated with an increase in LD cases.

### Meteorological factors and surface water with LD incidence

Despite the fact that surface water conditions such as compound concentrations were expected to be closely related to air temperature, total precipitation, and wind speed, no such associations were identified between the presence of any LD cases and those meteorological conditions within the combined model for the two. Surprisingly, there were indications that increases in the concentration of manganese and iron were positively associated and for aluminium negatively associated with the presence of LD cases in any amounts. Even more surprisingly, *given that cases are present*, there are indications that a further increase in the number of cases is associated with the *decrease* in temperature values and in wind speed, which was counterintuitive. For the river water compounds, *given that cases are present*, the increases in concentration of orthophosphate and aluminium are associated with the decrease in the number of cases, while the increases in the concentration of strontium are associated with the increase in the number of cases. Among the associations identified, *given that cases are present,* the strongest effect per compound unit increase was observed for the orthophosphate. No effect was observed just for the presence of LD cases. This finding is a bit counterintuitive, since phosphate can be a limiting factor that plays a role in buffering effect and as an essential nutrient for the growth and replication of bacteria in the natural environment, and when supplemented into drinking water, it can lead to a rapid increase in the concentration of heterotrophic bacteria [96]. After closer inspection (Supplementary Figure S7), the orthophosphate concentrations only had non-negative values starting from July 2004 up to January 2009. This time period is roughly within the ‘spiked’ period for cases (Figure 1 and Table 3), but the number of years with multiple LD case reports empirically looks to be longer (i.e., 2004 to 2009). We hypothesise that the non-negative orthophosphate concentration (i.e., presence of such) was only for a short subset of months (52 out of 228, Supplementary Figure S7), which could make the corresponding statistical estimates unstable. More research is recommended on the topic since there are direct lab indications that orthophosphates in drinking water promote *L. pneumophila* growth [97]. In particular, considering that *L. pneumophila* multiplies inside heterotrophic organisms [98], its concentration is likely the highest in surface water when the availability of total organic carbon and concentrations of heterotrophic bacteria is maximised during the late summer and early fall [99]. Also, inorganic phosphates can be key factors in metabolism and have been previously found to increase both the proliferation of bacteria and the species diversity within biofilms growing in simulated distribution systems, even in the presence of a disinfectant residual [100,101]. Due to the relative abundance of organic carbon in the surface waters of the Potomac River, phosphate is likely the limiting nutrient for bacterial growth in the surface water, which is dramatically increased during treatment, whereas organic carbon is actually reduced [102]. Either way, it is worth emphasising that the utilised predictors were measured in the surface river water, and even if such growth of *L. pneumophila* was or was not present, the linkage with the reported LD incidence may be dubious. People do not drink or otherwise consume river water directly.

From previous findings, there have been indications that changes in manganese concentrations are associated with the increase in the proliferation of *L. pneumophila* in hot water systems [103], while more research was recommended on the topic. Also, there has been evidence that the fluctuations in manganese concentrations that were associated with rainfall and water temperature could be the result of increased infiltration of soil containing manganese into the surface water during rainfall events, the higher solubility of manganese with increases in water temperature, or the increase in humic acid concentration from decaying organic matter washed into the river by rainfall [104]. The identified positive statistically significant associations between the increase in the concentration of manganese in surface (river) water and the increased odds of reporting any LD cases agree with those findings of the proliferation of *L. pneumophila* under such conditions. Also, *given that cases are present,* no such associations were detected for the counts (i.e., *log component*) of the model. This may be in part due to the relative sparsity of non-zero counts for most months (157 counts total presented in 71 months out of 228) and the inability to detect the statistically significant effect on the quantity with such a sample.

From the considered models, there were indications that high concentrations of aluminium were associated with the decrease in LD counts for *both* models (Poisson and negative binomial) and both components, that is, the *logit component* (any LD cases presence) and *log component* (changes in counts).

The other significant factors were the positive association with strontium for the *logit component* (any LD cases presence) for the Poisson model. It is worth noting that the estimates for iron were unstable for the Poisson-only version of the model, so the corresponding results should be interpreted with caution.

### Meteorological factors and treatment plant water with LD incidence

For the meteorological factors and the treatment plant water models, no meteorological factors for either *logit component* or *log components* of any of the models were statistically significant for the presence of any counts or increase in counts. Such findings are expected since after processing water characteristics are changing and the effect of the meteorological factors becomes more limited.

The other compounds whose increase in concentration was associated with a statistically significant change in LD incidence and LD counts included total organic carbon, turbidity, aluminium, chlorine, strontium, zinc, barium, nitrate, and orthophosphate.

In particular, though some of the changes in surface water quality can be explained by natural phenomena and (potential) weather and climatic changes, the presence of phosphates in the finished water has a more direct link to treatment practices. More specifically, during November 2000, the District of Columbia Water and Sewer Authority (DC Water or DCWASA) switched the primary disinfectant from free chlorine (Cl_2_) to chloramine (NH_2_Cl) by the addition of ammonia during the water treatment process [105]. It was not until 2002 that public health officials noticed elevated blood lead levels in DC children, leading to the discovery that the switch to chloramines had altered the chemistry of the municipal water, resulting in the leaching of lead from the service lines joining the distribution system to individual houses [106]. To reduce the lead concentrations and prevent corrosion of the distribution system, supplementation of the finished water with 2 to 4 ppm of zinc orthophosphate was started in mid-2004 [107]. Though it had been later documented that phosphate addition can lead to higher concentrations of bacteria in finished water and has previously been implicated in an outbreak of LD, it was uncertain what effect this would have on water quality [108,109]. Also, phosphates in the finished water can inhibit copper-silver ionisation systems that are commonly used by both nursing homes and hospital facilities as a secondary disinfection technique to protect immunocompromised individuals from water-borne nosocomial infections [110–112]. The precise scope of use of such copper-silver ionisation systems throughout Washington, DC, is unknown.

While the heterotrophic plate count predictor was not significant in both models, the turbidity parameter was highly significant in *both* models, which indicated associations of water turbidity concentration with LD incidence (Supplementary Figure S27). This water turbidity parameter should be amongst the focuses of potential future studies. The other important components identified from the analysis were chlorine presence and total organic carbon, which were both associated with LD incidence and require more focus in the future.

## Conclusions

The findings from this study support previous work identifying the seasonality of LD incidence. The majority of cases during the study period were observed in the month of June to December. The three months with the largest total numbers reported were August, October, and November. After accounting for seasonality and for the presence of LD cases, temperature increase was determined to be statistically significant in predicting the increase in the number of cases.

For the surface water, there were indications that an increase in concentration of manganese and iron was positively associated and of aluminium was negatively associated with the presence of LD cases in any amounts. Also, given that cases were present for the meteorological factors, there were indications that a further increase in the number of cases was associated with the decrease in temperature and wind speed. For the river water compounds, given that cases were present, the increase in concentrations of orthophosphate and aluminium was associated with the decrease in the number of cases, while the increase in the concentration of strontium was associated with the increase in the number of cases.

For the treatment plant water, there were indications that the increase in concentrations of total organic carbon, turbidity, aluminium, and chlorine was positively associated with the presence of LD cases in any amounts, while the increase in the concentration of strontium and zinc was negatively associated with the presence of LD cases in any amounts. For the treatment plant water compounds, given that cases were present, the increase in concentrations of barium and chlorine was associated with the increase in the number of cases, while the increase in the concentration of nitrate, orthophosphate, and turbidity was associated with the decrease in the number of cases.

More research is recommended on the relationships between the concentrations of phosphates and turbidity in drinking water and their relations to the LD incidence. Also, further research is recommended on the changes in concentrations of water compounds which are identified as statistically significant in this study based on the meteorological factors which could affect them. Understanding these relationships can contribute to raising people’s prevention awareness during peak times and calling for government and public health scientists’ attention to water quality, therefore reducing future LD outbreaks within the United States.

## Data Availability

The entire analysis was performed in R programming language and the source code together with the analyzed data were made publicly available on GitHub and can be downloaded [81].

## Supplementary material

For supplementary material accompanying this paper visit http://doi.org/10.1017/S0950268823000651. click here to view supplementary material
