# Supplementary material for: An investigation of the seasonal relationships between meteorological factors, water quality, and sporadic cases of Legionnaires’ disease in Washington, DC
Source: Epidemiol Infect. 2023 May 15;151:e88. doi: 10.1017/S0950268823000651 (PMC10265736; doi:10.1017/S0950268823000651)
Supplement: Supplementary file 1 [file S0950268823000651sup001.pdf]

An Investigation of the Seasonal Relationships Between Meteorological Factors, Water Quality, and Sporadic Cases of Legionnaires' Disease in Washington, DC (Supplement)

Alexander Kirpich[1,*], Aleksandr Shishkin[1,+], Pema Lhewa[1,+] , Chen Yang[2,+]

Michael E. von Fricken[3], Michael H. Norris[4,5], Thomas A. Weppelmann[6]

[1]Department of Population Health Sciences, School of Public Health, Georgia State University, Atlanta, Georgia, United States of America

[2]Department of Epidemiology, Harvard T.H. Chan School of Public Health, Harvard University, Boston, Massachusetts, United States of America

[3]Department of Global and Community Health, College of Health and Human Services George Mason University, Fairfax, Virginia, United States of America

[4]Department of Geography, University of Florida, Gainesville, Florida, United States of America

[5]Emerging Pathogens Institute, University of Florida, Gainesville, Florida, United States of America

[6]Department of Internal Medicine, University of South Florida, Tampa, Florida, United States of America

Key Words: Legionnaires' disease, drinking water, environmental water quality, meteorological factors

+Equal Contribution: Those authors contributed equally.

***Corresponding Author**: Alexander Kirpich (akirpich@gsu.edu)

Table S1. The complete list of the river water compounds used for the analysis together with the corresponding measurement units.

**Variable**	**Unit**
pH	
Nitrate	ppm
Orthophosphate	ppm
Manganese	ppb
Strontium	ppb
Barium	ppb
Nickel	ppb
Total Organic Carbon	ppb
Turbidity	NTU
Aluminum	ppb
Total Coliform	MPN/100mL
Zinc	ppb
Iron	ppb

Table S2. The complete list of the treatment plant water compounds used for the analysis together with the corresponding measurement units.

**Variable**	**Unit**
pH	
Nitrate	ppm
Orthophosphate	ppm
Manganese	ppb
Strontium	ppb
Barium	ppb
Nickel	ppb
Total Organic Carbon	ppb
Turbidity	NTU
Aluminum	ppb
Total Coliform (Positive)	MPN/100mL
Heterotrophic Plate Count	CFU/mL
Zinc	ppb
Iron	ppb
Chlorine	ppm

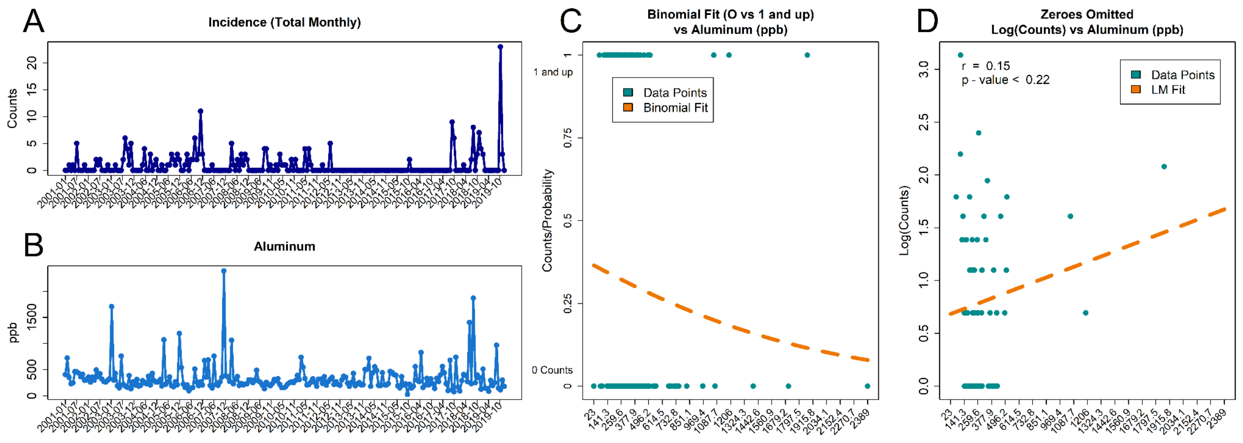

Figure S1. The summaries of the A) reported incidence and B) river Aluminum (ppb). The logistic model fit for zero (coded as 0) vs non-zero (code as 1) counts is provided in panel C. Counts on the log scale i.e. Log(Counts) vs river Aluminum (ppb) are provided in panel C. The panel C also contains the linear regression fitted line of Log(Counts) vs river Aluminum. The corresponding linear regression statistics (correlation estimate and p-value for the statistical test of slope to be zero) are also provided.

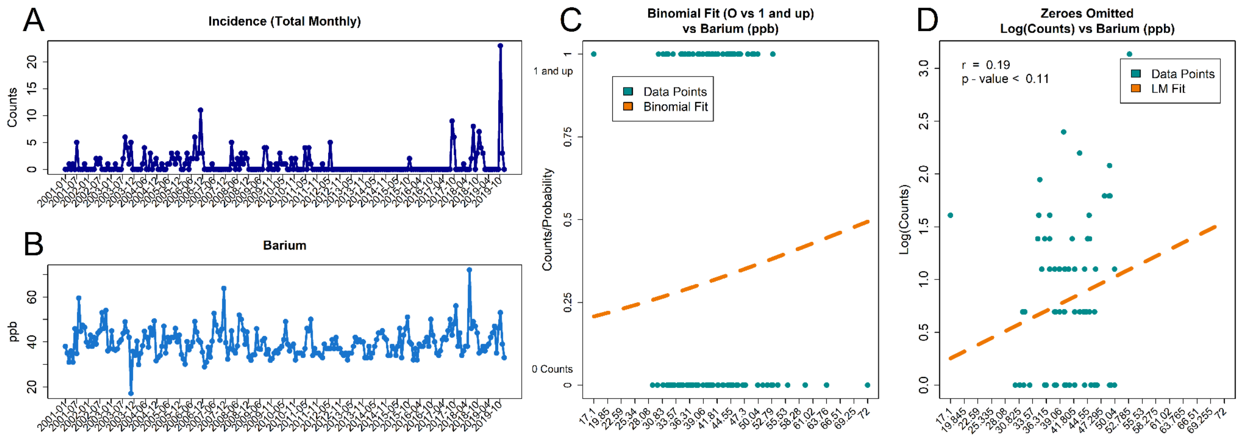

Figure S2. The summaries of the A) reported incidence and B) river Barium (ppb). The logistic model fit for zero (coded as 0) vs non-zero (code as 1) counts is provided in panel C. Counts on the log scale i.e. Log(Counts) vs river Barium (ppb) are provided in panel C. The panel C also contains the linear regression fitted line of Log(Counts) vs river Barium. The corresponding linear regression statistics (correlation estimate and p-value for the statistical test of slope to be zero) are also provided.

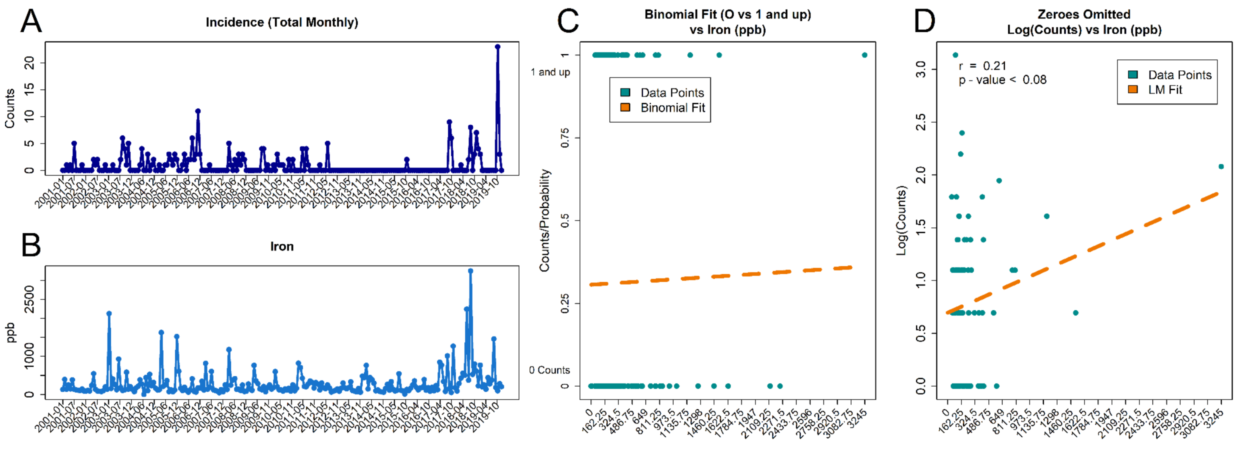

Figure S3. The summaries of the A) reported incidence and B) river Iron (ppb). The logistic model fit for zero (coded as 0) vs non-zero (code as 1) counts is provided in panel C. Counts on the log scale i.e. Log(Counts) vs river Iron (ppb) are provided in panel C. The panel C also contains the linear regression fitted line of Log(Counts) vs river Iron. The corresponding linear regression statistics (correlation estimate and p-value for the statistical test of slope to be zero) are also provided.

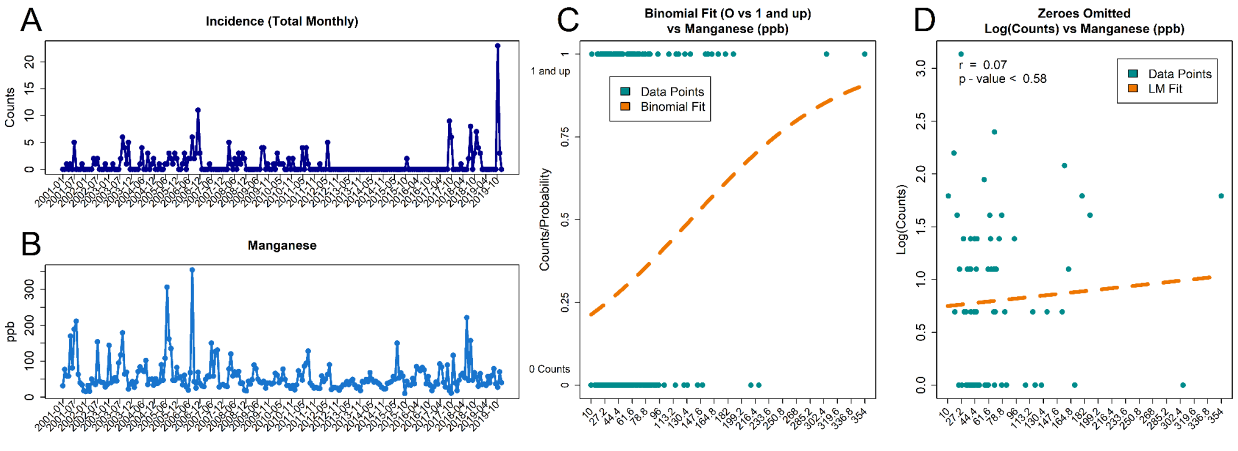

Figure S4. The summaries of the A) reported incidence and B) river Manganese (ppb). The logistic model fit for zero (coded as 0) vs non-zero (code as 1) counts is provided in panel C. Counts on the log scale i.e. Log(Counts) vs river Manganese (ppb) are provided in panel C. The panel C also contains the linear regression fitted line of Log(Counts) vs river Manganese. The corresponding linear regression statistics (correlation estimate and p-value for the statistical test of slope to be zero) are also provided.

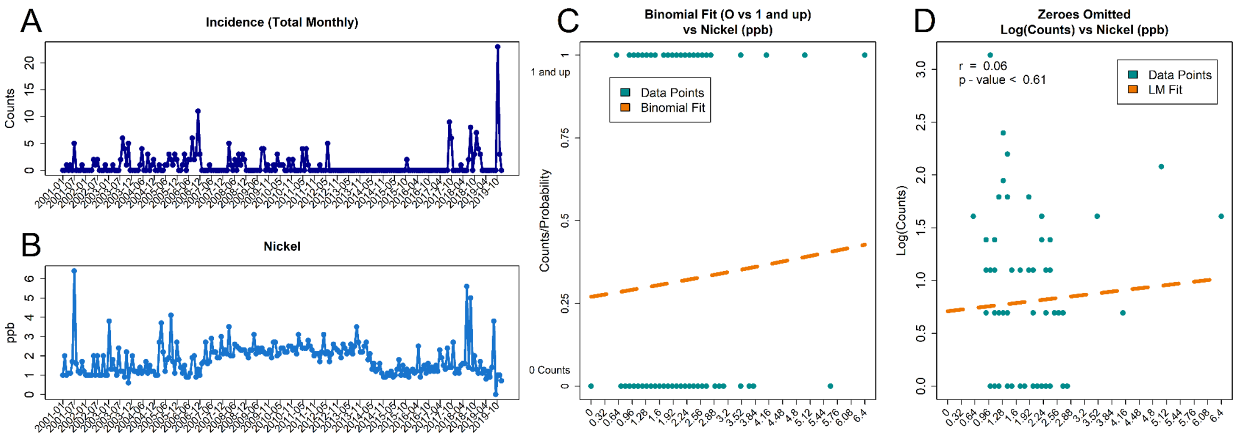

Figure S5. The summaries of the A) reported incidence and B) river Nickel (ppb). The logistic model fit for zero (coded as 0) vs non-zero (code as 1) counts is provided in panel C. Counts on the log scale i.e. Log(Counts) vs river Nickel (ppb) are provided in panel C. The panel C also contains the linear regression fitted line of Log(Counts) vs river Nickel. The corresponding linear regression statistics (correlation estimate and p-value for the statistical test of slope to be zero) are also provided.

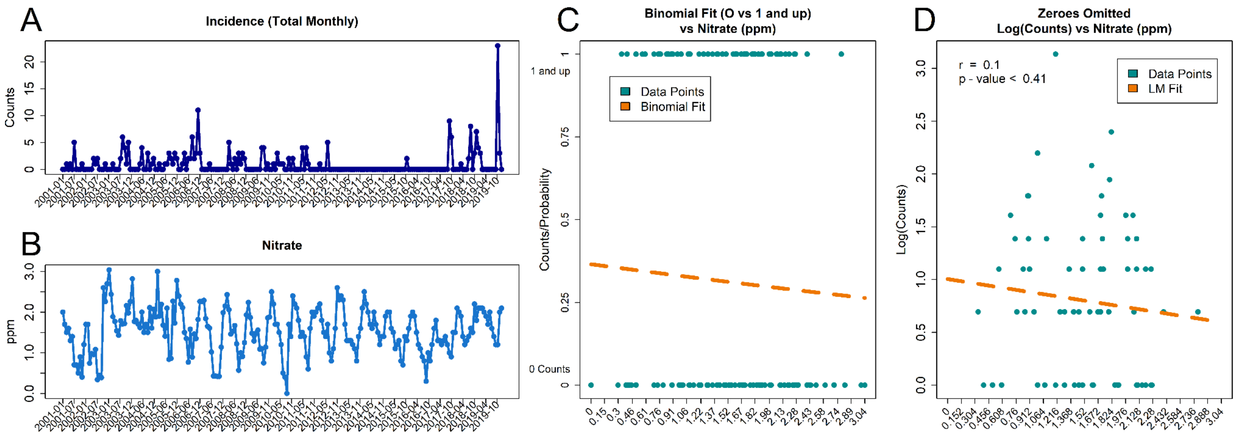

Figure S6. The summaries of the A) reported incidence and B) river Nitrate (ppm). The logistic model fit for zero (coded as 0) vs non-zero (code as 1) counts is provided in panel C. Counts on the log scale i.e. Log(Counts) vs river Nitrate (ppm) are provided in panel C. The panel C also contains the linear regression fitted line of Log(Counts) vs river Nitrate. The corresponding linear regression statistics (correlation estimate and p-value for the statistical test of slope to be zero) are also provided.

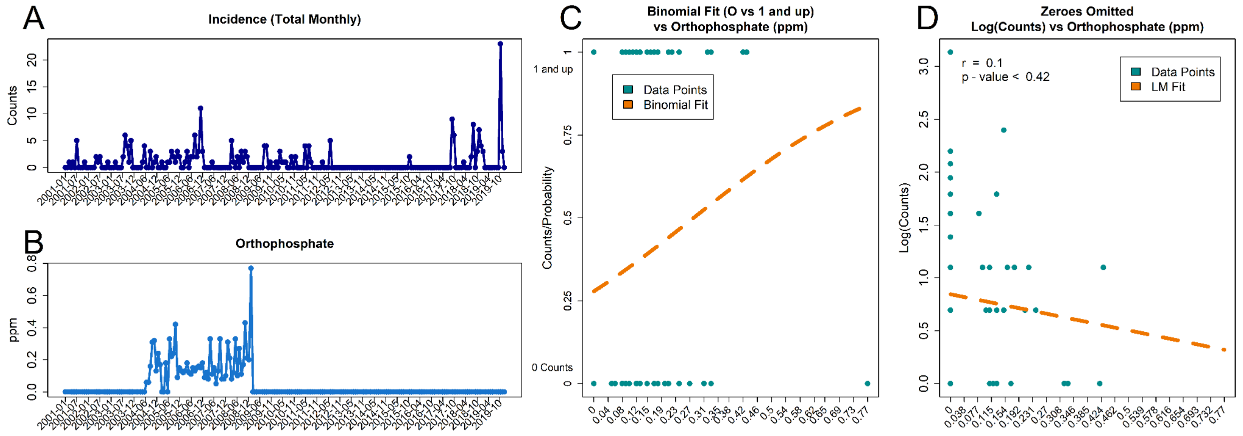

Figure S7. The summaries of the A) reported incidence and B) river Orthophosphate (ppm). The logistic model fit for zero (coded as 0) vs non-zero (code as 1) counts is provided in panel C. Counts on the log scale i.e. Log(Counts) vs river Orthophosphate (ppm) are provided in panel C. The panel C also contains the linear regression fitted line of Log(Counts) vs river Orthophosphate. The corresponding linear regression statistics (correlation estimate and p-value for the statistical test of slope to be zero) are also provided.

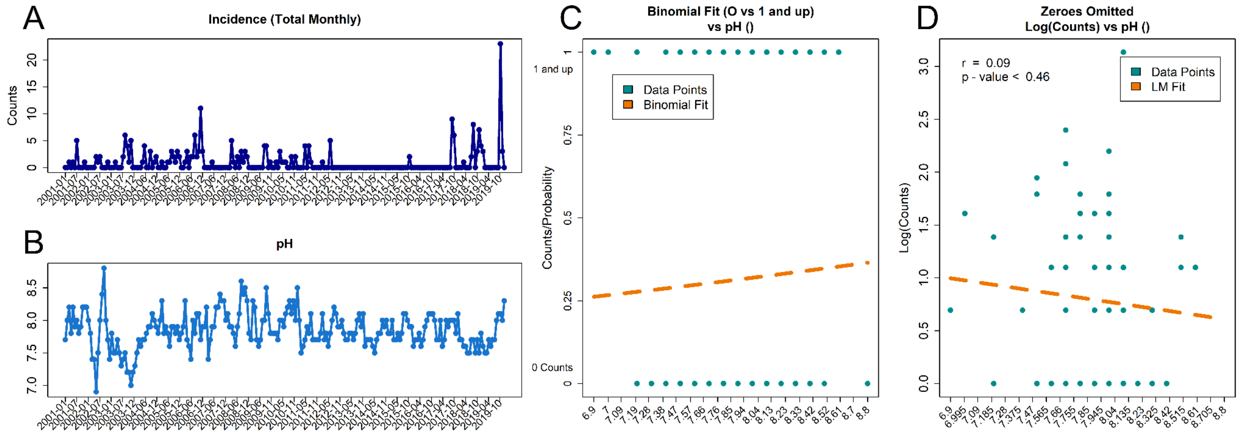

Figure S8.The summaries of the A) reported incidence and B) river pH (). The logistic model fit for zero (coded as 0) vs non-zero (code as 1) counts is provided in panel C.Counts on the log scale i.e. Log(Counts) vs river pH () are provided in panel C.The panel C also contains the linear regression fitted line of Log(Counts) vs river pH. The corresponding linear regression statistics (correlation estimate and p-value for the statistical test of slope to be zero) are also provided.

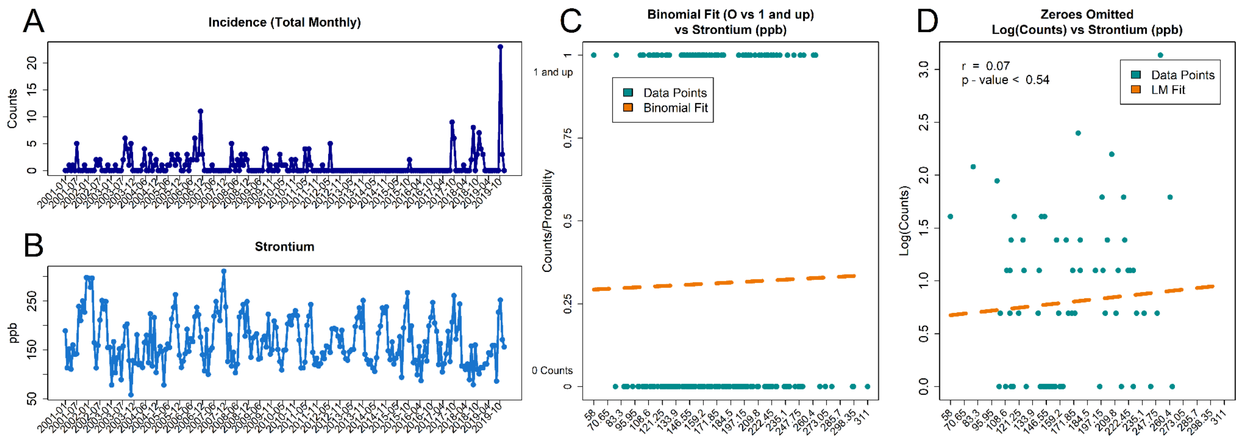

Figure S9. The summaries of the A) reported incidence and B) river Strontium (ppb). The logistic model fit for zero (coded as 0) vs non-zero (code as 1) counts is provided in panel C. Counts on the log scale i.e. Log(Counts) vs river Strontium (ppb) are provided in panel C. The panel C also contains the linear regression fitted line of Log(Counts) vs river Strontium. The corresponding linear regression statistics (correlation estimate and p-value for the statistical test of slope to be zero) are also provided.

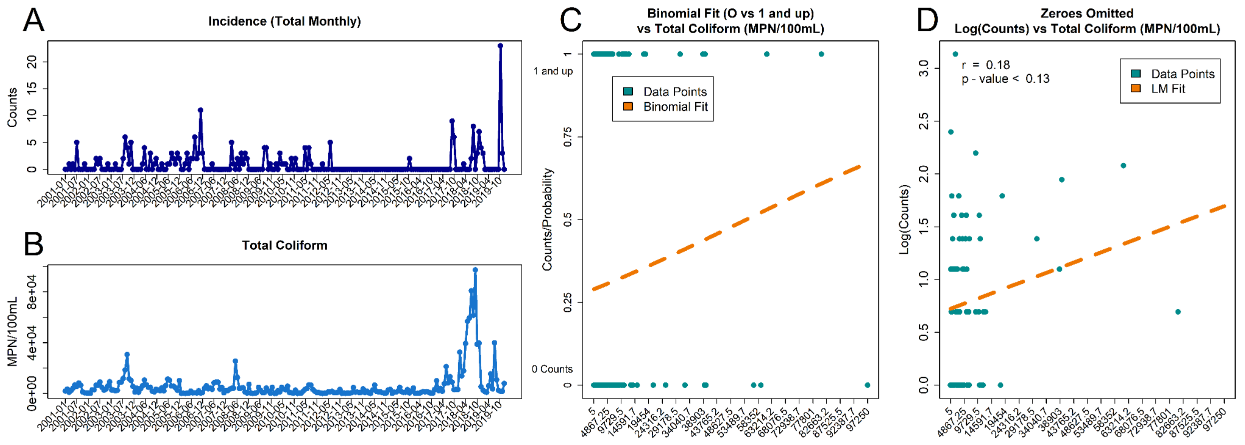

Figure S10. The summaries of the A) reported incidence and B) river Total Coliform (MPN/100mL). The logistic model fit for zero (coded as 0) vs non-zero (code as 1) counts is provided in panel C. Counts on the log scale i.e. Log(Counts) vs river Total Coliform (MPN/100mL) are provided in panel C. The panel C also contains the linear regression fitted line of Log(Counts) vs river Total Coliform. The corresponding linear regression statistics (correlation estimate and p-value for the statistical test of slope to be zero) are also provided.

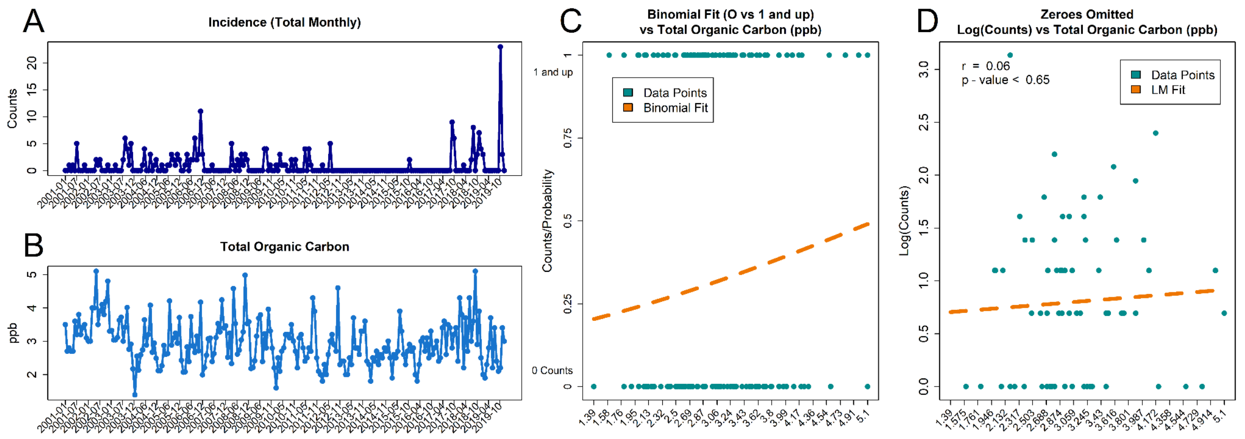

Figure S11. The summaries of the A) reported incidence and B) river Total Organic Carbon (ppb). The logistic model fit for zero (coded as 0) vs non-zero (code as 1) counts is provided in panel C. Counts on the log scale i.e. Log(Counts) vs river Total Organic Carbon (ppb) are provided in panel C. The panel C also contains the linear regression fitted line of Log(Counts) vs river Total Organic Carbon. The corresponding linear regression statistics (correlation estimate and p-value for the statistical test of slope to be zero) are also provided.

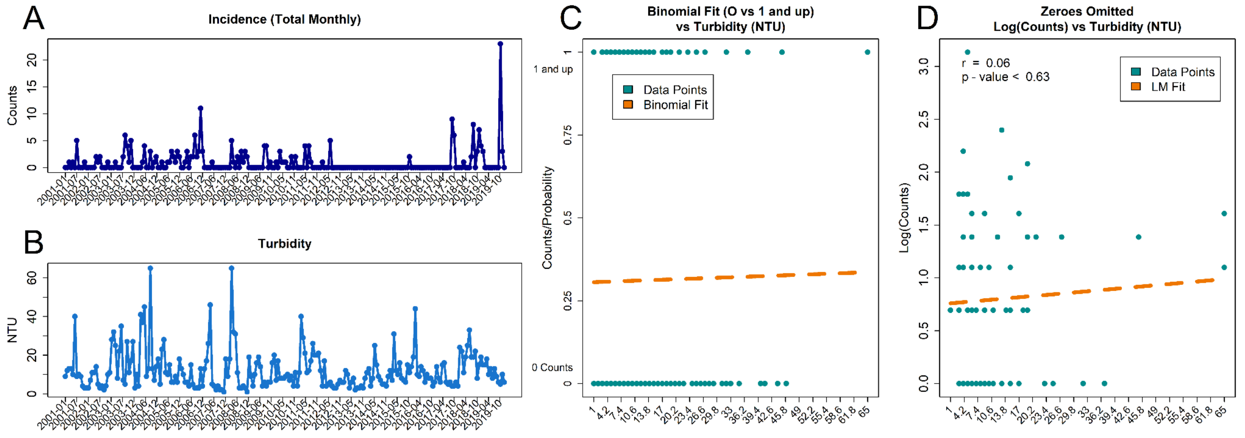

Figure S12. The summaries of the A) reported incidence and B) river Turbidity (NTU). The logistic model fit for zero (coded as 0) vs non-zero (code as 1) counts is provided in panel C. Counts on the log scale i.e. Log(Counts) vs river Turbidity (NTU) are provided in panel C. The panel C also contains the linear regression fitted line of Log(Counts) vs river Turbidity. The corresponding linear regression statistics (correlation estimate and p-value for the statistical test of slope to be zero) are also provided.

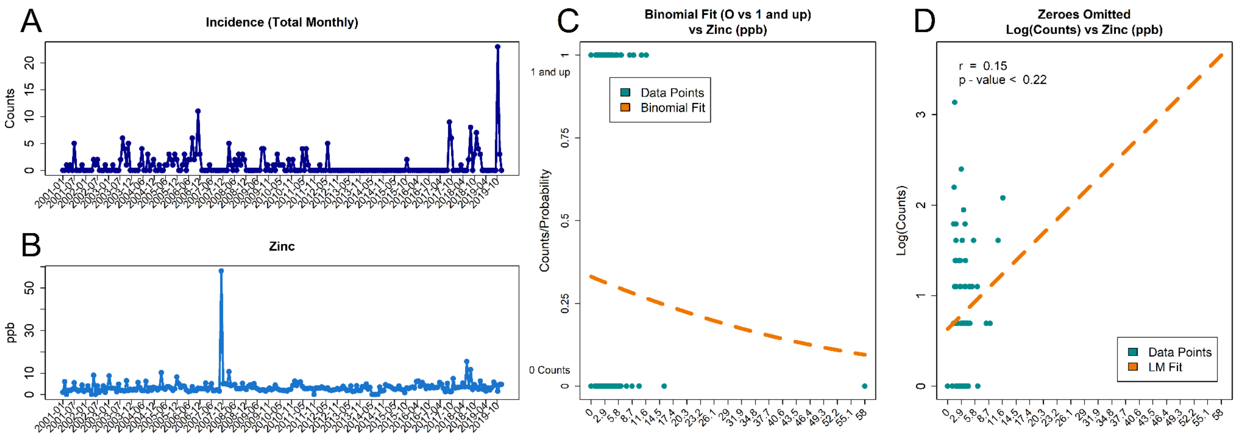

Figure S13. The summaries of the A) reported incidence and B) river Zinc (ppb). The logistic model fit for zero (coded as 0) vs non-zero (code as 1) counts is provided in panel C. Counts on the log scale i.e. Log(Counts) vs river Zinc (ppb) are provided in panel C. The panel C also contains the linear regression fitted line of Log(Counts) vs river Zinc. The corresponding linear regression statistics (correlation estimate and p-value for the statistical test of slope to be zero) are also provided.

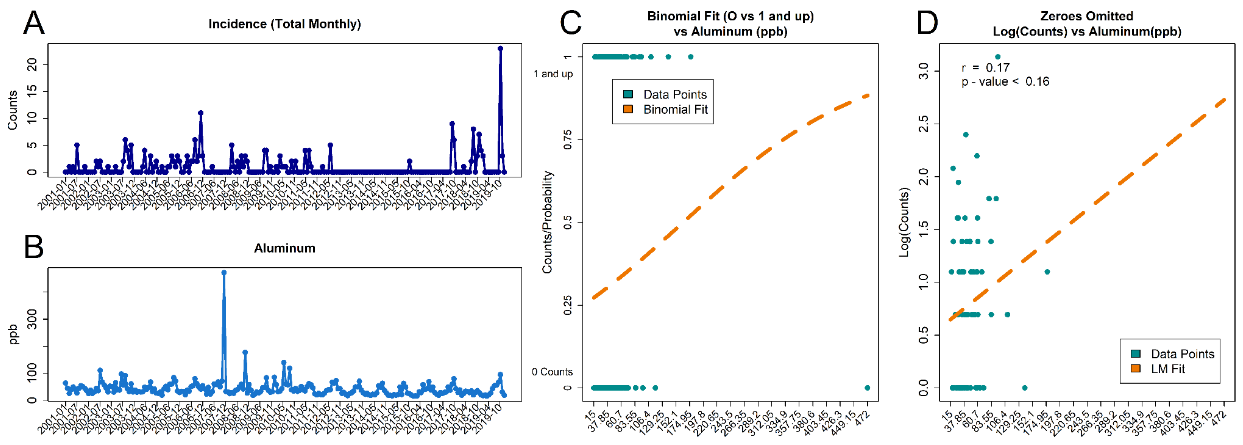

Figure S14. The summaries of the A) reported incidence and B) treatment plant Aluminum (ppb). The logistic model fit for zero (coded as 0) vs non-zero (code as 1) counts is provided in panel C. Counts on the log scale i.e. Log(Counts) vs treatment plant Aluminum (ppb) are provided in panel C. The panel C also contains the linear regression fitted line of Log(Counts) vs river Aluminum. The corresponding linear regression statistics (correlation estimate and p-value for the statistical test of slope to be zero) are also provided.

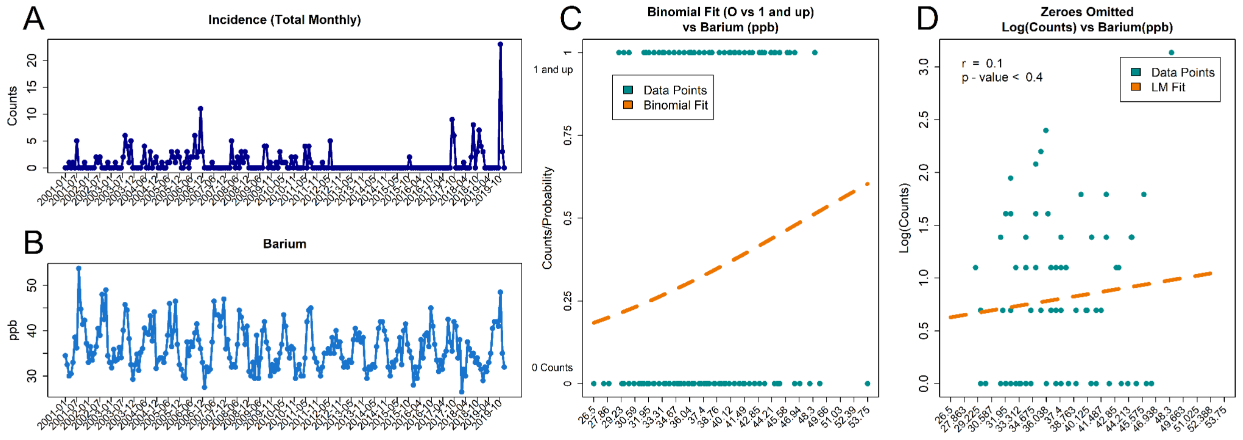

Figure S15. The summaries of the A) reported incidence and B) treatment plant Barium (ppb). The logistic model fit for zero (coded as 0) vs non-zero (code as 1) counts is provided in panel C. Counts on the log scale i.e. Log(Counts) vs treatment plant Barium (ppb) are provided in panel C. The panel C also contains the linear regression fitted line of Log(Counts) vs river Barium. The corresponding linear regression statistics (correlation estimate and p-value for the statistical test of slope to be zero) are also provided.

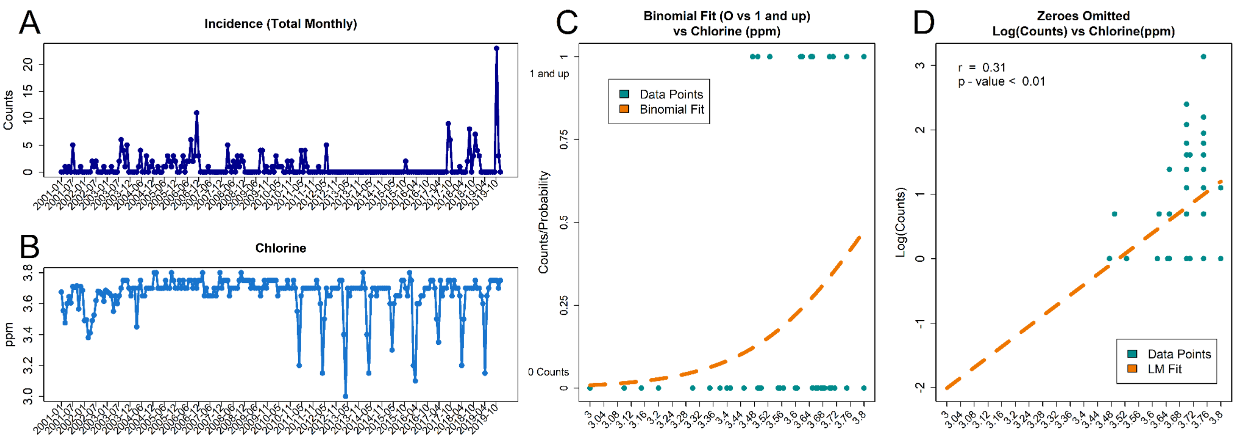

Figure S16. The summaries of the A) reported incidence and B) treatment plant Chlorine (ppm). The logistic model fit for zero (coded as 0) vs non-zero (code as 1) counts is provided in panel C. Counts on the log scale i.e. Log(Counts) vs treatment plant Chlorine (ppm) are provided in panel C. The panel C also contains the linear regression fitted line of Log(Counts) vs river Chlorine. The corresponding linear regression statistics (correlation estimate and p-value for the statistical test of slope to be zero) are also provided.

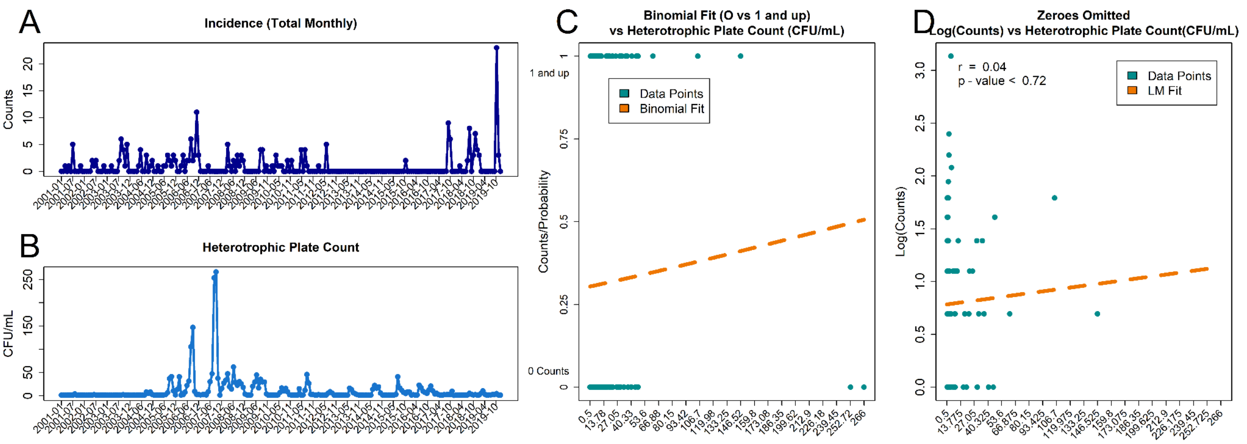

Figure S17. The summaries of the A) reported incidence and B) treatment plant HPC (ppb). The logistic model fit for zero (coded as 0) vs non-zero (code as 1) counts is provided in panel C. Counts on the log scale i.e. Log(Counts) vs treatment plant HPC (ppb) are provided in panel C. The panel C also contains the linear regression fitted line of Log(Counts) vs river HPC. The corresponding linear regression statistics (correlation estimate and p-value for the statistical test of slope to be zero) are also provided.

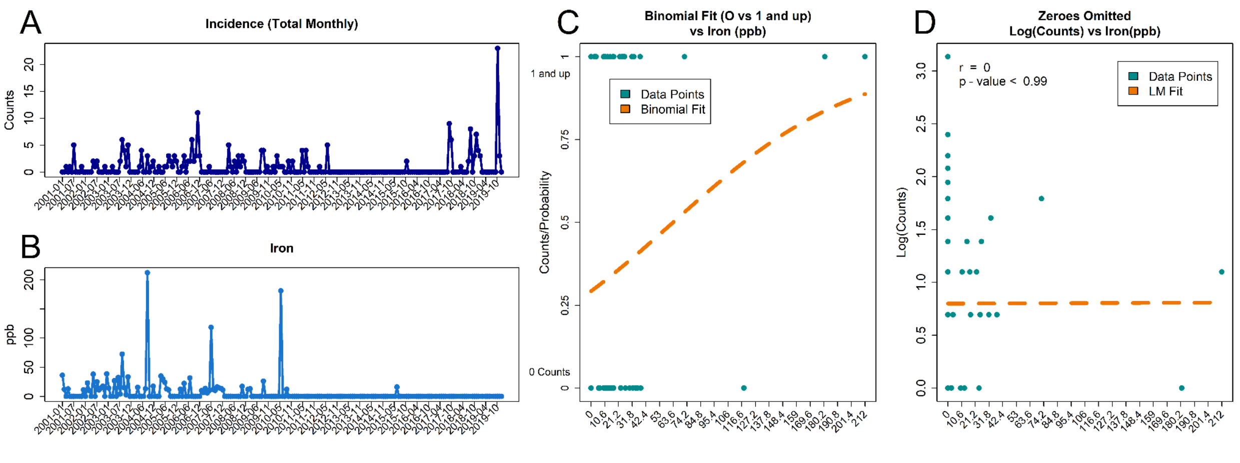

Figure S18. The summaries of the A) reported incidence and B) treatment plant Iron (ppb). The logistic model fit for zero (coded as 0) vs non-zero (code as 1) counts is provided in panel C. Counts on the log scale i.e. Log(Counts) vs treatment plant Iron (ppb) are provided in panel C. The panel C also contains the linear regression fitted line of Log(Counts) vs river Iron. The corresponding linear regression statistics (correlation estimate and p-value for the statistical test of slope to be zero) are also provided.

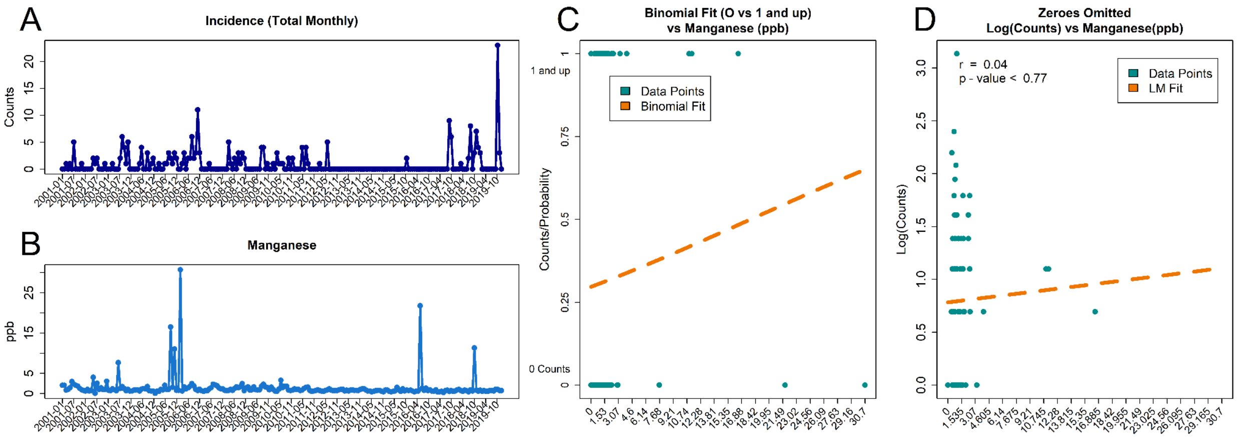

Figure S19. The summaries of the A) reported incidence and B) treatment plant Manganese (ppb). The logistic model fit for zero (coded as 0) vs non-zero (code as 1) counts is provided in panel C. Counts on the log scale i.e. Log(Counts) vs treatment plant Manganese (ppb) are provided in panel C. The panel C also contains the linear regression fitted line of Log(Counts) vs river Manganese. The corresponding linear regression statistics (correlation estimate and p-value for the statistical test of slope to be zero) are also provided.

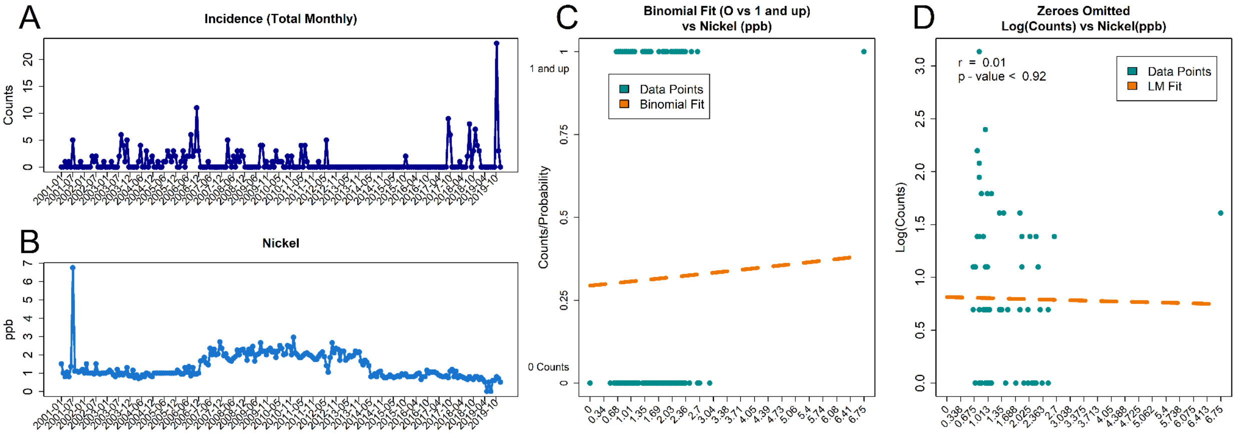

Figure S20. The summaries of the A) reported incidence and B) treatment plant Nickel (ppb). The logistic model fit for zero (coded as 0) vs non-zero (code as 1) counts is provided in panel C. Counts on the log scale i.e. Log(Counts) vs treatment plant Nickel (ppb) are provided in panel C. The panel C also contains the linear regression fitted line of Log(Counts) vs river Nickel. The corresponding linear regression statistics (correlation estimate and p-value for the statistical test of slope to be zero) are also provided.

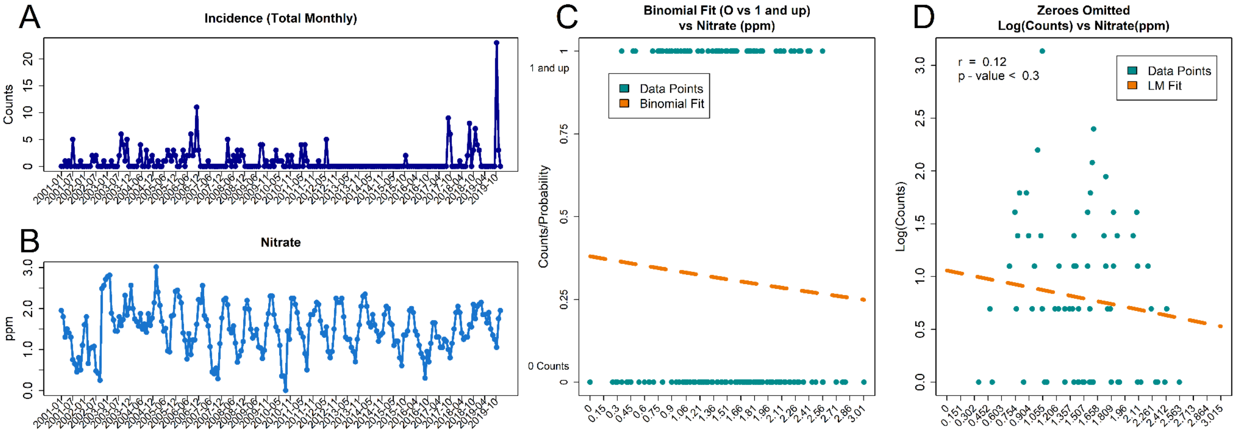

Figure S21. The summaries of the A) reported incidence and B) treatment plant Nitrate (ppm). The logistic model fit for zero (coded as 0) vs non-zero (code as 1) counts is provided in panel C. Counts on the log scale i.e. Log(Counts) vs treatment plant Nitrate (ppm) are provided in panel C. The panel C also contains the linear regression fitted line of Log(Counts) vs river Nitrate. The corresponding linear regression statistics (correlation estimate and p-value for the statistical test of slope to be zero) are also provided.

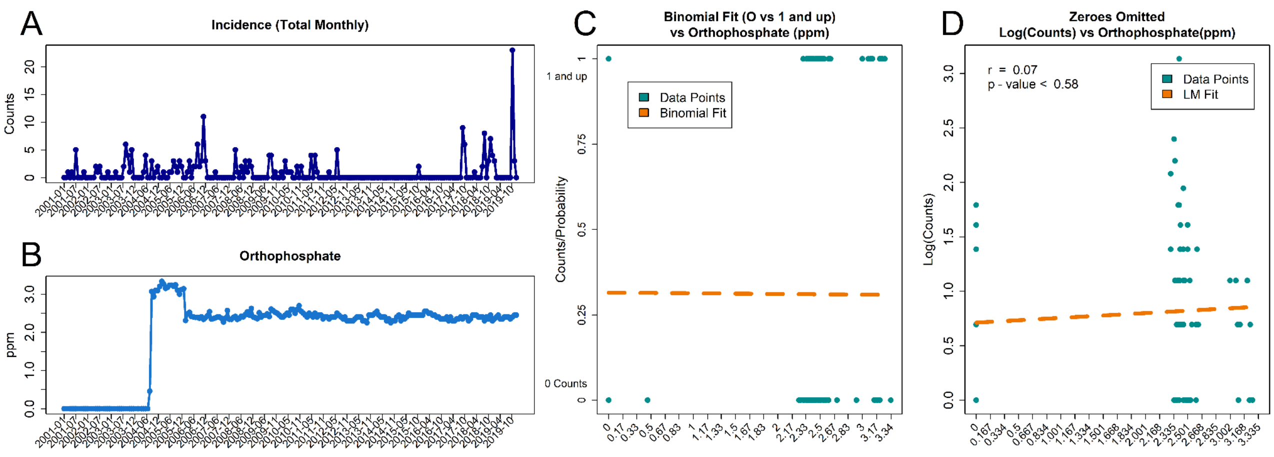

Figure S22. The summaries of the A) reported incidence and B) treatment plant Orthophosphate (ppm). The logistic model fit for zero (coded as 0) vs non-zero (code as 1) counts is provided in panel C. Counts on the log scale i.e. Log(Counts) vs treatment plant Orthophosphate (ppm) are provided in panel C. The panel C also contains the linear regression fitted line of Log(Counts) vs river Orthophosphate. The corresponding linear regression statistics (correlation estimate and p-value for the statistical test of slope to be zero) are also provided.

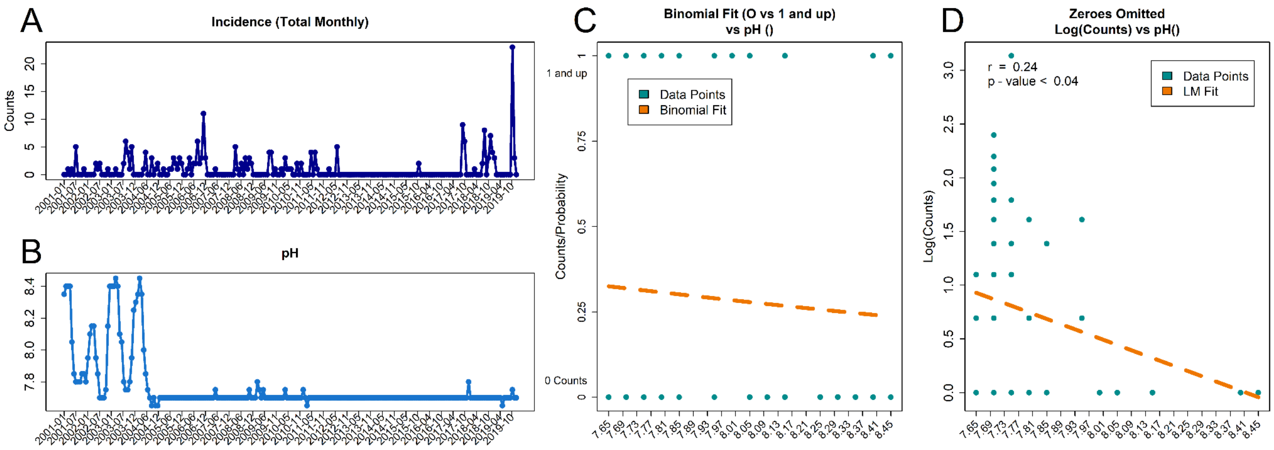

Figure S23. The summaries of the A) reported incidence and B) treatment plant pH (). The logistic model fit for zero (coded as 0) vs non-zero (code as 1) counts is provided in panel C. Counts on the log scale i.e. Log(Counts) vs treatment plant pH () are provided in panel C. The panel C also contains the linear regression fitted line of Log(Counts) vs river pH. The corresponding linear regression statistics (correlation estimate and p-value for the statistical test of slope to be zero) are also provided.

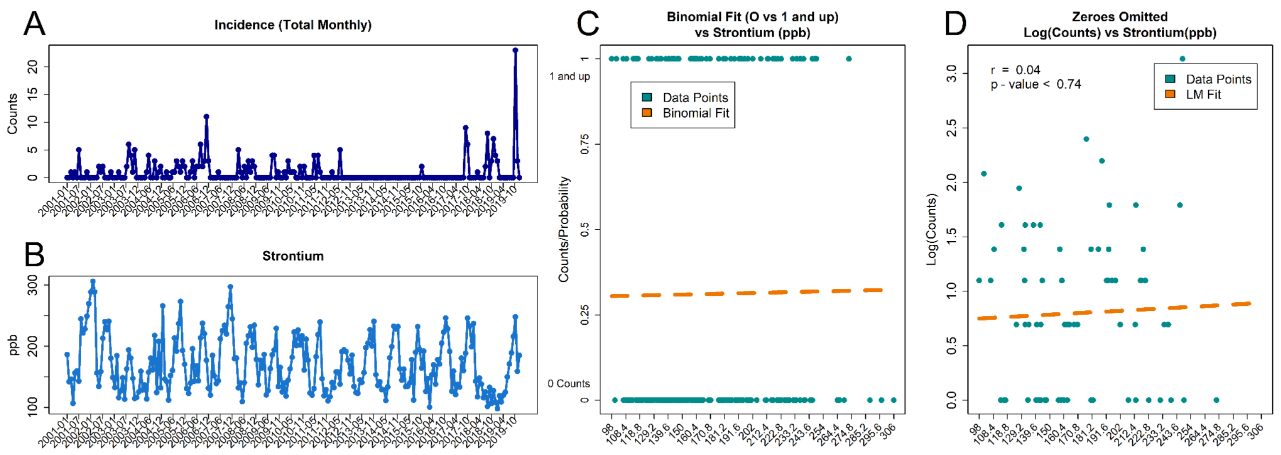

Figure S24. The summaries of the A) reported incidence and B) treatment plant Strontium (ppb). The logistic model fit for zero (coded as 0) vs non-zero (code as 1) counts is provided in panel C. Counts on the log scale i.e. Log(Counts) vs treatment plant Strontium (ppb) are provided in panel C. The panel C also contains the linear regression fitted line of Log(Counts) vs river Strontium. The corresponding linear regression statistics (correlation estimate and p-value for the statistical test of slope to be zero) are also provided.

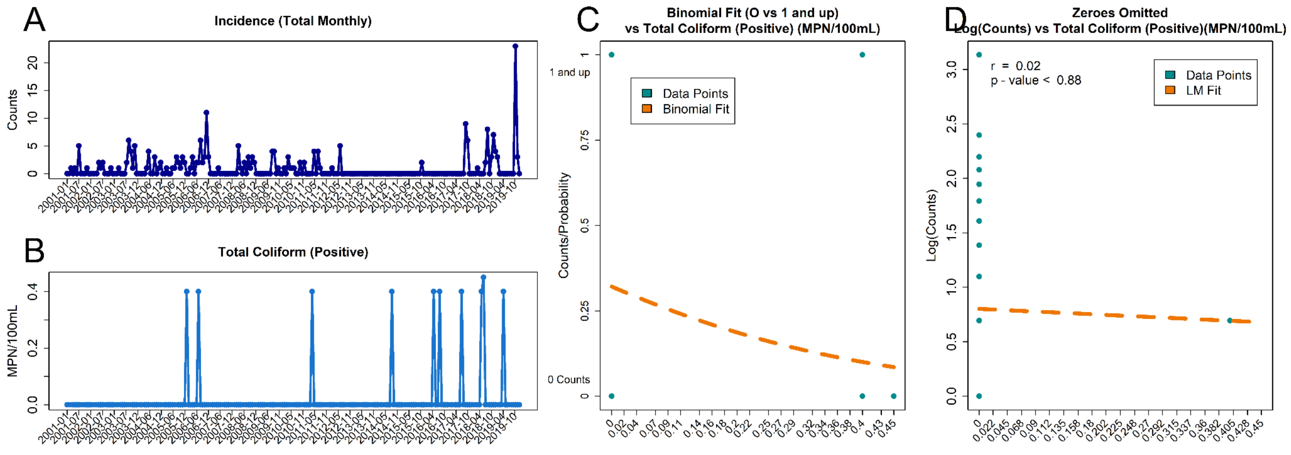

Figure S25. The summaries of the A) reported incidence and B) treatment plant Total Coliform (Positive) (MPN/100mL). The logistic model fit for zero (coded as 0) vs non-zero (code as 1) counts is provided in panel C. Counts on the log scale i.e. Log(Counts) vs treatment plant Total Coliform (Positive) (MPN/100mL) are provided in panel C. The panel C also contains the linear regression fitted line of Log(Counts) vs river Total Coliform (Positive). The corresponding linear regression statistics (correlation estimate and p-value for the statistical test of slope to be zero) are also provided.

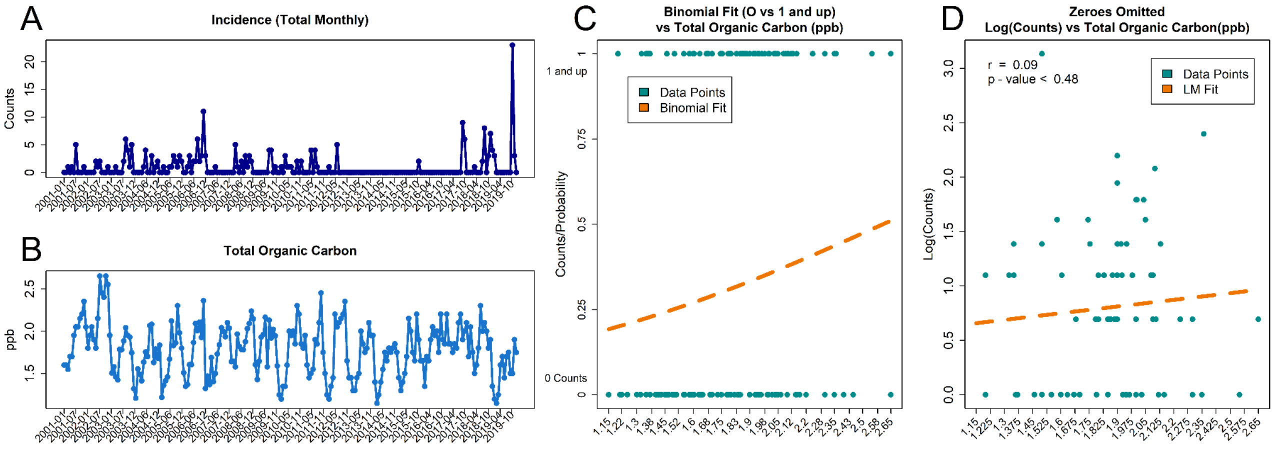

Figure S26. The summaries of the A) reported incidence and B) treatment plant Total Organic Carbon (ppb). The logistic model fit for zero (coded as 0) vs non-zero (code as 1) counts is provided in panel C. Counts on the log scale i.e. Log(Counts) vs treatment plant Total Organic Carbon (ppb) are provided in panel C. The panel C also contains the linear regression fitted line of Log(Counts) vs river Total Organic Carbon. The corresponding linear regression statistics (correlation estimate and p-value for the statistical test of slope to be zero) are also provided.

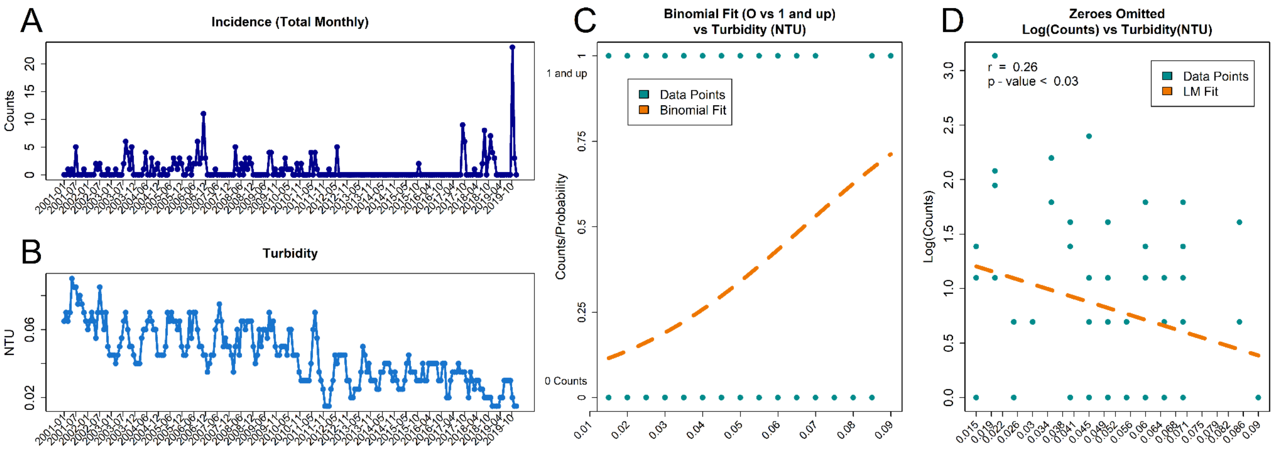

Figure S27. The summaries of the A) reported incidence and B) treatment plant Turbidity (NTU). The logistic model fit for zero (coded as 0) vs non-zero (code as 1) counts is provided in panel C. Counts on the log scale i.e. Log(Counts) vs treatment plant Turbidity (NTU) are provided in panel C. The panel C also contains the linear regression fitted line of Log(Counts) vs river Turbidity. The corresponding linear regression statistics (correlation estimate and p-value for the statistical test of slope to be zero) are also provided.

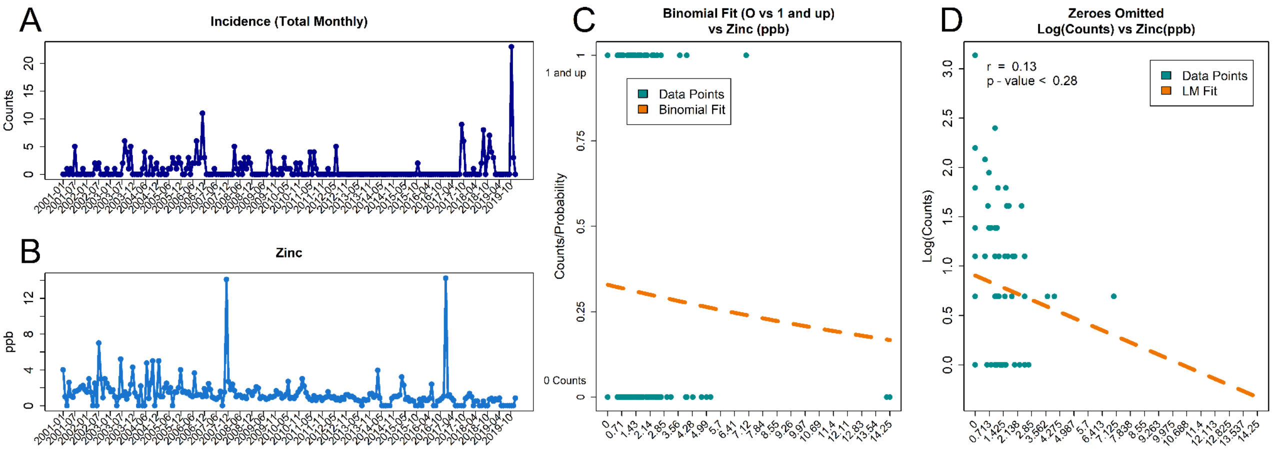

Figure S28. The summaries of the A) reported incidence and B) treatment plant Zinc (ppb). The logistic model fit for zero (coded as 0) vs non-zero (code as 1) counts is provided in panel C. Counts on the log scale i.e. Log(Counts) vs treatment plant Zinc (ppb) are provided in panel C. The panel C also contains the linear regression fitted line of Log(Counts) vs river Zinc. The corresponding linear regression statistics (correlation estimate and p-value for the statistical test of slope to be zero) are also provided.
